# Phenotypic Heterogeneity Analysis of *APC*-Mutant Colon Cancer by Proteomics and Phosphoproteomics Identifies RAI14 as a Key Prognostic Determinant in East Asians and Westerners

**DOI:** 10.1016/j.mcpro.2023.100532

**Published:** 2023-03-18

**Authors:** Rou Zhang, Meng Hu, Hai-Ning Chen, Xiuxuan Wang, Zhili Xia, Yu Liu, Rui Wang, Xuyang Xia, Yang Shu, Dan Du, Wenbo Meng, Shiqian Qi, Yuan Li, Heng Xu, Zong-Guang Zhou, Lunzhi Dai

**Affiliations:** 1 State Key Laboratory of Biotherapy, National Clinical Research Center for Geriatrics and General Practice Medical Center, West China Hospital, Sichuan University, Chengdu, China; 2 Department of General Surgery, Colorectal Cancer Center, West China Hospital, Sichuan University, Chengdu, China; 3 The First Clinical Medical College, Lanzhou University, Lanzhou, China; 4 Research Core Facility, Advanced Mass Spectrometry Center, Frontiers Science Center for Disease-Related Molecular Network, West China Hospital, Sichuan University, Chengdu, China; 5 West China-Washington Mitochondria and Metabolism Centre, Institutes for Systems Genetics, West China Hospital, Sichuan University, Chengdu, China; 6 Institute of Digestive Surgery, West China Hospital, Sichuan University, Chengdu, China

**Keywords:** Colon cancer, Adenomatous polyposis coli, Multi-omics, Phenotypic heterogeneity, RAI14

## Abstract

Adenomatous polyposis coli (*APC*) is an important tumor suppressor and is mostly linked to the regulation of the Wnt/β-catenin signaling pathway. *APC* mutation has been identified as an early event in more than 80% of sporadic colorectal cancers (CRCs). Moreover, prognostic differences are observed in CRC patients with *APC* mutations. Although previous genomics studies have investigated the roles of concomitant gene mutations in determining the phenotypic heterogeneity of *APC*-mutant tumors, valuable prognostic determinants for *APC*-mutant CRC patients are still lacking. Based on the proteome and phosphoproteome data, we classified *APC*-mutant colon cancer patients and revealed genomic, proteomic, and phosphoproteomic heterogeneity in *APC*-mutant tumors. More importantly, we identified RAI14 as a key prognostic determinant for *APC*-mutant but not *APC*-wildtype colon cancer patients. The heterogeneity and the significance of prognostic biomarkers in *APC*-mutant tumors were further validated in the Clinical Proteomic Tumor Analysis Consortium (CPTAC) colon cancer cohort. In addition, we found that colon cancer patients with high expression of RAI14 were less responsive to chemotherapy. Knockdown of RAI14 in cell lines led to reduced cell migration and changes in epithelial-mesenchymal transition (EMT)–related markers. Mechanistically, knockdown of RAI14 remodeled the phosphoproteome associated with cell adhesion, which might affect EMT marker expression and promote F-actin degradation. Collectively, this work describes the phenotypic heterogeneity of *APC*-mutant tumors and identifies RAI14 as an important prognostic determinant for *APC*-mutant colon cancer patients. The prognostic utility of RAI14 in *APC*-mutant colon cancer will provide early warning and increase the chance of successful treatment.

## Highlights

• An atlas of genome, proteome, and phosphoproteome of CRC is provided.
• Integrative proteomic and phosphoproteomic analysis reveals the molecular heterogeneity in APC-MUT CRC.
• RAI14 is a key prognostic determinant for APC-MUT CRC patients.
• High levels of RAI14 suggest decreased response to chemotherapy and increased cell migration.

## Graphical Abstract

**Figure:**
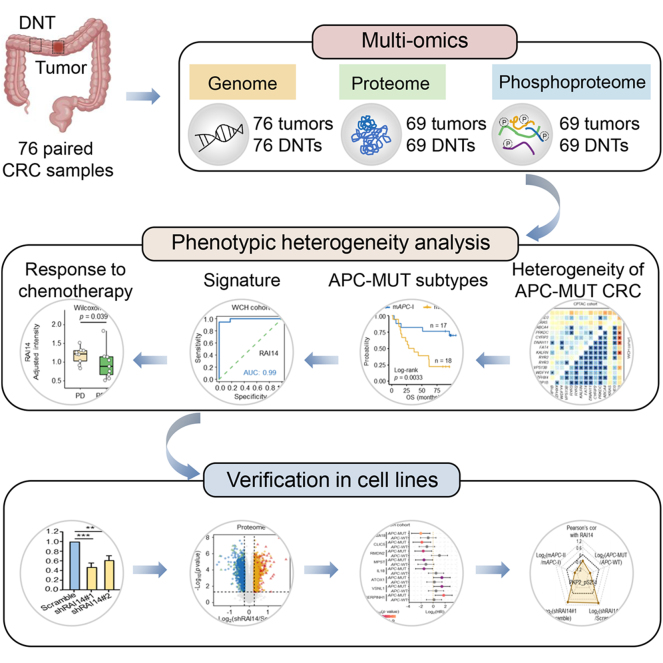

## In Brief

The prognostic phenotype is heterogeneous in colorectal cancer (CRC) patients with APC mutations, while the diagnostic biomarkers remain largely unknown. Here, we described the phenotypic heterogeneity of APC-mutant tumors and identified RAI14 as a key prognostic determinant for APC-mutant colon cancer patients. The prognostic utility of RAI14 in APC-mutant colon cancer will provide early warning and increase the chance of successful treatment.

Adenomatous polyposis coli (*APC*) is an important tumor suppressor and is most directly related to the regulation of the Wnt/β-catenin signaling pathway (1). It induces β-catenin degradation and keeps β-catenin signaling in the OFF state (2) and is involved in a wide range of cellular processes, such as DNA repair, cytoskeleton stabilization, cell differentiation, and apoptosis (3). Inactivation of *APC* promotes nuclear accumulation of β-catenin and may trigger colorectal cancer (CRC) (4, 5). *APC* gene mutation has been identified as an early event in more than 80% of sporadic CRCs (6, 7). Over 90% of *APC* mutations in CRC do not result in complete loss of APC protein but instead produce stable truncated gene products without β-catenin–binding sites, nuclear localization sequences, SAMP repeats, or a C-terminal basic region (8). The functions of these stable APC truncations are very complex, and some of them even gain new functions (9, 10, 11), leading to the phenotypic heterogeneity of *APC*-mutant (*APC*-MUT) tumors.

Thus far, the molecular basis for predicting the overall survival (OS) of *APC*-MUT CRC patients is largely based on genomics (12, 13). The ideal goal is to use mutation fingerprints to identify *APC*-MUT CRC patients with unfavorable prognosis, which may help to personalize treatment plans for these patients. However, the construction of mutation fingerprints is very complex, because different mutations in an identical gene may cause phenotypic heterogeneity (14). Furthermore, even the same gene mutation can lead to distinct phenotypes because of genetic background effects (15), which further add difficulties in predicting phenotypic outcomes. Of note, heterogeneity analysis of *APC*-MUT CRC shows that tumors with wildtype (WT) *APC* are more malignant than those with a single *APC* mutation, whereas tumors with two *APC* mutations and concomitant *KRAS* and *TP53* mutations make patients have the worst survival (13). Despite some progress has been made, the molecular characteristics of *APC*-MUT tumors remain less understood, and valuable prognostic determinants for *APC*-MUT CRC patients are still lacking.

The combination of omics technologies and molecular classification methods has been widely used to help elucidate the heterogeneity of cancer (16, 17, 18, 19). Inspired by these achievements, in this study, we carried out genomics of 76 pairs of colon cancer tissues and proteomics and phosphoproteomics analysis of 69 pairs of colon cancer tissues, of which 35 patients identified mutated *APC*s. Molecular classification based on the proteome and phosphoproteome data demonstrated the heterogeneity in *APC*-MUT tumors and identified RAI14 as a key prognostic marker. These findings were validated in the Clinical Proteomic Tumor Analysis Consortium (CPTAC) colon cancer cohort (20). In addition, we found that increased RAI14 promoted *APC*-MUT cancer cell migration by remodeling the cell adhesion–associated phosphoproteome, suggesting that RAI14 and related regulators are potential targets for treating malignant *APC*-MUT tumors.

## Experimental Procedures

### Sample Collection and Preparation

A total of 76 paired colon cancer samples were obtained from West China Hospital (WCH) with informed consent and the approval of the Research Ethics Committee (Permission number: 2020(374)). The research was conducted in accordance with the Declaration of Helsinki ethical principles. The inclusion criteria recruited patients undergoing primary surgery without any anticancer preoperative therapy for colon cancer. Clinical information, including age, gender, tumor site, and tumor node metastasis (TNM) stage, was collected. Patients were followed-up with a median time of 62 months. OS begins from surgery and finishes at death or the last follow-up visit. Tissues were snap-frozen in liquid nitrogen and then stored at −80 °C for long-term storage. Two pathologists from the Department of Pathology of WCH of Sichuan University examined the pathological specimens independently.

### Proteomic and Phosphoproteomic Analyses

#### Protein Extraction and Digestion

Tissue or cell samples were shredded and lysed in modified RIPA buffer (NaCl 150 mM, 50 mM Tris (pH = 7.5), Triton X-100 1% (v/v), Na-deoxycholate 0.05% (w/v), sodium deoxycholate 0.01% (w/v), 1% cocktail (v/v), 1% phosphatase inhibitors (v/v)), and the procedure “protein 01. 01” gentleMACS Dissociator (Miltenyi Biotec GmbH) was used to homogenize the tissue samples. Then, the crude lysates were sonicated for 5 min (model JY92-IIN, 227.5 W, 3 s on followed by 10 s off), followed by 20 min of centrifugation (20,000*g*, 4 °C). The supernatant was transferred into a new tube as a whole extract, and the protein concentration was measured using the Bradford protein assay (Bio-Rad). Approximately, 100 μg protein lysates of each sample were subsequently reduced with 10 mM tris(2-carboxyethyl) phosphine at 56 °C for 1 h, alkylated with 20 mM iodoacetamide for 30 min to block the free cysteine residues in the dark at room temperature, and precipitated with methanol/chloroform/water (CH_3_OH: CHCl_3_: H_2_O = 4:1:3). The proteins were then digested by trypsin for 12 h at a 1:50 (w/w, trypsin/protein) ratio.

#### TMT-10 Labeling of Peptides

Anhydrous acetonitrile (ACN) was used to dissolve the tandem mass tag (TMT) 10-plex Isobaric Label Reagent (Thermo Fisher Scientific) after equilibrating to room temperature before use. Peptides digested by trypsin were labeled with TMT reagents according to the manual. Then, 5% hydroxylamine was added to quench the unreacted TMT reagents. Peptides labeled by different TMT labels were mixed and concentrated to dryness.

#### Peptide Fractionation

For proteomics, TMT-labeled peptides were fractionated by reversed-phase HPLC under basic conditions after desalting. A flow rate of 1 ml/min was set to separate samples by using mobile phase buffer A (98% H_2_O, 2% ACN, pH = 10) and mobile phase buffer B (90% ACN, 10% H_2_O, pH = 10). A standard 120 min LC gradient run was as follows: 0 to 10 min, 3% buffer B; 10 to 85 min, 3% to 35% buffer B; 85 to 95 min, 35% to 60% buffer B; 95 to 105 min, 60% to 100% buffer B; 105 to 120 min, 100% to 3% buffer B. The resulting 120 fractions were then combined into 15 fractions (colon cancer tissues) or 30 fractions (cells) and then vacuum-centrifuged to dryness. After being desalted with C18 ZipTip, the peptides were analyzed by LC-MS/MS. For phosphorylomics, the mixture was first divided into 15 (colon cancer tissues) or 21 fractions (cells) by a C18 solid-phase extraction cartridge (100 mg packing material) and then combined into five fractions and seven fractions to dryness.

#### The Enrichment of Phosphorylated Peptides

PureCube Fe-NTA Agarose Beads (Cube Biotech) were used to enrich the phosphopeptides. In brief, 20 μl of Fe-NTA agarose beads were used to enrich the phosphopeptides of each sample. After being washed with washing buffer three times, the Fe-NTA agarose beads were incubated with the desalting peptides in 300 μl washing buffer for 1 h with a 3D shaker at room temperature. After being washed with washing buffer five times, 150 μl elution buffer (50% ACN, 2.5% ammonia) was added to elute the peptides. Eight microliters of 20% TFA were used to neutralize the eluate. The peptides were concentrated to dryness and analyzed by LC-MS/MS after desalting with C18 ZipTip.

#### LC-MS/MS Analysis in Orbitrap Exploris 480

After being desalted with C18 ZipTip, the peptides were dried in vacuum and resuspended in buffer A (2% ACN, 0.1% formic acid (FA)). The resuspended peptides were loaded on a 75 μm (inner diameter) × 30 cm (length) analytical column that was pulled and packed in-house with C18 particles (DIKMA). A Nano EASY-nL 1200 (Thermo Fisher Scientific) coupled to an Orbitrap Eclipse 480 mass spectrometer (Thermo Fisher Scientific) was used to perform LC-MS/MS analysis. A 65 min gradient from 4% to 100% buffer B (80% ACN and 0.1% FA) was set to analyze the peptide samples, and then the flow rate of 300 nl/min was set to perform the data-dependent acquisition in positive ion mode. Mass spectrometry (MS) spectra were acquired from 350 to 1800 m/z with a resolution of 60,000 at m/z = 200, and the full MS automatic gain control (AGC) target was set to 300%. For MS/MS scans, the isolation window was set as 0.7 m/z and fragmented with a normalized collision energy (NCE) of 36%. Precursor ions with charge states of z = 1 or 8 or unassigned charge states were excluded.

#### LC-MS/MS Analysis in Q Exactive HF-X

After being desalted with C18 ZipTip, the peptides were dried in vacuum and resuspended in buffer A (2% ACN, 0.1% FA). The resuspended peptides were loaded on a 75 μm (inner diameter) × 2 cm (length) trap column and a 75 μm (inner diameter) × 25 cm (length) analytical column, which was pulled and packed in-house with C18 particles (DIKMA). A Nano EASY-nL 1200 (Thermo Fisher Scientific) coupled to a Q Exactive HF-X mass spectrometer (Thermo Fisher Scientific) was used to perform LC-MS/MS analysis. A 65 min gradient from 12% to 100% (proteomics) or 6% to 100% (phosphoproteomics) buffer B (80% ACN and 0.1% FA) was set to analyze the peptide samples, and then the flow rate of 330 nl/min was set to perform the data-dependent acquisition in positive ion mode. MS spectra were acquired from 350 to 1600 m/z with a resolving power of 60,000 at m/z = 200. The AGC value was set to 3.0e^6^, and the maximum fill time was 100 ms. For MS/MS scans, the top 20 most intense parent ions were selected with an isolation window of 0.6 m/z and fragmented with an NCE of 30% (proteomics) or stepped NCEs of 25% and 31% (phosphoproteomics). Precursor ions with charge states of z = 1 or 8 or unassigned charge states were excluded.

#### LC-MS/MS Analysis in Q Exactive Plus

After being desalted with C18 ZipTip, the peptides were dried in vacuum and resuspended in buffer A (2% ACN, 0.1% FA). The resuspended peptides were loaded on a 75 μm (inner diameter) × 2 cm (length) trap column and a 75 μm (inner diameter) × 25 cm (length) analytical column, which was pulled and packed in-house with C18 particles (DIKMA). A Nano EASY-nL 1000 (Thermo Fisher Scientific) coupled to a Q Exactive Plus mass spectrometer (Thermo Fisher Scientific) was used to perform LC-MS/MS analysis. A 90 min gradient from 14% to 100% buffer B (80% ACN and 0.1% FA) was set to analyze the peptide samples, and then the flow rate of 330 nl/min was set to perform the data-dependent acquisition in positive ion mode. MS spectra were acquired from 350 to 1600 m/z with a resolving power of 60,000 at m/z = 200. The AGC value was set to 3.0e^6^, and the maximum fill time was 100 ms. For MS/MS scans, the top 20 most intense parent ions were selected with an isolation window of 0.6 m/z and fragmented with a stepped NCE of 25% and 31%. Precursor ions with charge states of z = 1 or 8 or unassigned charge states were excluded.

#### MS Database Searching

MaxQuant (version 1.6) was used to search the raw files against the Swiss-Prot human protein sequence database (https://www.uniprot.org/, updated on 01/2017; 20,413 protein sequences). The database contains all protein sequences in reverse order and sequences from common contaminant proteins (21). The mass tolerance of the precursor peptide was 10 ppm, and the mass tolerance of the fragment ion was 0.02 Da. Two missed trypsin cleavages were allowed. Precursor intensity fraction 75% filtered quantification was reported using min, and the minimum amino acid length was set to 6. TMT on lysine residues and peptide N termini and cysteine carbamidomethylation were set as fixed modifications, while oxidation of methionine and protein N-terminal acetylation were set as variable modifications. At the protein and peptide levels, proteins with a false discovery rate <1% were included. For phosphoproteomics, phosphorylation (S/T/Y) was set as a dynamic modification.

### Experimental Design and Statistical Rationale

A total of 76 treatment-naive colon cancer patients with paired distant normal tissues (DNTs) were collected from the WCH (referred to as the WCH cohort). A schematic diagram of the experimental design is presented in supplemental Fig. S1. Whole-exome sequencing (WES) was performed on all paired samples to detect any possible genetic variants in the cancer genome. Proteomic and phosphoproteomic analyses were conducted on 69 paired samples which were grouped into 18 TMT batches, respectively. The TMT labeling information is provided in supplemental Table S1. Clean global proteome data were obtained with R (version 4.2.1). Specifically, nonunique peptides and peptides identified as potential contaminants or reverse sequences were first removed. Next, proteins with fewer than two identified unique peptides were excluded. The protein intensity was calculated from the sum of its unique peptide intensity. The total protein abundance of each sample in the same batch was adjusted to the same level. To eliminate noise interference, the protein intensity of tumor or DNT was divided by the protein intensity of the common reference sample to obtain protein sample-to-reference (S/R) values. The S/R values were used for calculating the adjusted intensities (T/N) of proteins, which were log2-transformed for subsequent analyses. Then, samples and proteins from 18 batches were consolidated into a single matrix. Zero values were replaced by “NA”. Proteins present in over 50% of samples in both the APC-MUT and APC-WT subgroups were reserved and imputed by the “rf” method using the R package “mice”. For global phosphoproteome data cleaning, a similar approach was used with R (version 4.2.1). Peptides derived from potential contaminants or reverse sequences were first removed. The total phosphopeptide abundance of each sample in the same batch was adjusted to the same level. The phosphopeptide intensity of the tumor or DNT was divided by the phosphopeptide intensity of the common reference sample to obtain S/R values. The S/R values were used for calculating the adjusted intensities (T/N) of phosphopeptides. Additionally, the T/N values were log2-transformed for subsequent analyses. Zero values were replaced by “NA”. Phosphopeptides present in over 50% of samples in both the APC-MUT and APC-WT subgroups were reserved and imputed by the “rf” method using the R package “mice”. Proteins with ratios of mAPC-II/mAPC-I >2 (or <0.5) and *p* values <0.05 (Wilcoxon rank-sum test) were defined as significantly differentially expressed proteins in mAPC-II subtype, and phosphosites with ratios of mAPC-II/mAPC-I >1.5 (or <0.667) and *p* values <0.05 (Wilcoxon rank-sum test) were defined as significantly differentially expressed phosphosites in mAPC-II subtype. Proteins and phosphosites with ratios of shRAI14/Scramble >1.2 (or <0.833) and *p* values <0.05 (Student’s *t* test) were defined as significantly differentially expressed proteins or phosphosites in cell lines. The Wilcoxon rank-sum test and Student’s *t* test were utilized to identify differential proteins and phosphosites in colon cancer tissues and cell lines, respectively. The DAVID database (https://david.ncifcrf.gov/) (22, 23) and Metascape database (http://metascape.org) (24) were applied to identify enriched biological pathways. The OS was the time calculated from diagnosis to dead or censored.

### Whole-Exome Sequencing

WES analysis was applied to 76 paired tumors and DNTs from colon cancer patients in the WCH cohort. Genomic DNA was quantified by a Qubit DNA Assay Kit in Qubit 2.0 Fluorometer (Invitrogen). An Agilent SureSelect Human All Exon kit (Agilent Technologies) was used to generate WES libraries according to the manufacturer’s recommendations. The DNA library was sequenced with an Illumina NovaSeq 6000 System, and 150 bp paired-end reads were generated. The original fluorescence image files obtained from the HiSeq platform were transformed to raw data by base calling and then converted to FASTQ format, which contains sequence information and corresponding sequencing quality information. All downstream bioinformatics analyses were based on high-quality clean data.

### Collection of Public Datasets

Publicly available multiomics data and clinical annotations of CRC samples were collected from six cohorts. Among them, one cohort was obtained from LinkedOmics (http://linkedomics.org/cptac-colon/) (25), and the other cohorts were collected from Bioportal (https://www.cbioportal.org/datasets) (26, 27).

### Somatic Copy Number Alteration Analysis

WES-derived BAM files processed in the somatic mutation detection pipeline were used in somatic copy number alteration analysis (SCNA). Focal-level, gene-level, and arm-level SCNAs were identified by Genomic Identification of Significant Targets in Cancer (GISTIC 2.0, version 6.15.28, https://cloud.genepattern.org) (28) to obtain significantly gained or lost SCNA regions (Q value < 0.1). To exclude false positives, thresholds were used with the following parameters: refgene file = Human_Hg19.mat, focal length cutoff = 0.50, gene gistic = yes, confidence level = 0.99. Other parameters were set as default.

### Mutational Signature Analysis

The somatic mutation signatures were extracted by a non-negative matrix factorization approach (29) with the R package “maftools”. The somatic mutational spectrum was obtained by the 96 combinations of six single base substitutions (C > A, C > G, C > T, T > A, T > C, and T > G) and their preceding and following bases. The extracted mutation signatures were defined by comparison to 49 known COSMIC cancer signatures (30). The coefficients of each sample calculated by the non-negative matrix factorization method were considered the contribution for mutation signatures.

### Univariate Survival Analysis

Survival curves were generated using the Kaplan–Meier method of specific variables of interest using the R package “survminer”. Hazard ratios and their 95% confidence intervals were estimated using the “coxph” function with the R package “Survival”. The log-rank test was applied to calculate differences between the patient groups.

### Consensus Clustering Analysis

Prior to clustering analysis, proteins and phosphosites that were present in at least 50% of the samples in both the *APC*-MUT and *APC*-WT subpopulations were imputed using the R package “mice” with the imputation parameters method = “rf”, m = 5, maxit = 5, and seed = 1234. To classify the *APC*-MUT colon cancer patients, the imputed datasets from the proteome and phosphoproteome were consolidated to a list, which was further subjected to unsupervised consensus clustering performed with the function “ExecuteCC” of the R package “ConsensusClusterPlus” (31). The imputation parameters were set as follows: maxK = 10, reps = 500, clusterAlg = “hc”, and distance = “pearson”. The higher silhouette coefficients with larger sample sizes of clusters were considered a preferred cluster result.

### Variable Selection Analysis

Variable selection analysis for *APC*-MUT colon cancer was performed by the function ‘‘VSURF’’ in the R package ‘‘VSURF’’ with a random forest (RF) algorithm. The number of trees was set to 10,000 (32). Mean importance was obtained for each protein and phosphosite, and the top 15 most important variables were subjected to subsequent analyses.

### Cross Validation of RF-Based Machine Learning Models

To build a cross validation between WCH data and CPTAC data, the two proteins FETUB and DAAM1 were excluded from top 15 important variables due to missing values in more than 50% of the CPTAC data. The correlation among 13 included important variables was calculated by the function “cor”, and the variables whose pair-wise Pearson’s correlation coefficients >0.9 were removed. Next, the linear correlation analysis between 13 variables was calculated by the function “findLinearCombos” of R package “caret”, and the variables detected with linear correlation were removed. Then, on the one hand, 101 RF-based models were built using WCH data through 10-fold cross validation by function “train” and validated using CPTAC data. One hundred one ROC curves were displayed, and 101 AUC values were calculated by function “roc”. On the other hand, 101 RF-based models were built using CPTAC data through 10-fold cross validation by function “train” and validated using WCH data. One hundred one ROC curves were displayed, and 101 AUC values were calculated by function “roc”.

### Western Blotting Analysis

First, RIPA buffer (NaCl 150 mM, 50 mM Tris (pH = 7.5), Triton X-100 1% (v/v), Na-deoxycholate 0.05% (w/v), sodium deoxycholate 0.01% (w/v), 1% cocktail (v/v), 1% phosphatase inhibitors (v/v)) was used to lyse the tissues or cells. After centrifugation, the Bradford assay was used to measure the protein concentrations. The prepared protein samples were separated by 10% or 12% SDS-PAGE and then transferred onto polyvinylidene fluoride membranes. The membranes were blocked with 5% nonfat dry milk in PBST and then incubated with anti-N-cadherin antibody (Proteintech, 22018-1-AP) (1:1000), anti-vimentin antibody (Proteintech, 10366-1-AP) (1:1000), anti-RAI14 antibody (Proteintech, 17507-1-AP) (1:1000), or anti-GAPDH antibody (Proteintech, 60004-1-Ig) (1:5000) overnight at 4 °C. Then, after washing three times with PBST, the membranes were incubated with the secondary antibody at room temperature for 1 h. Band images were detected using Immobilon Western HRP Substrate (Millipore).

### Cell Lines and Cell Culture

The human colon cancer cell lines DLD-1 and SW480 were obtained from the Cell Bank/Stem Cell Bank, Chinese Academy of Sciences. Dulbecco’s modified Eagle’s medium (#C11995-065, Gibco) and RPMI 1640 medium (1640) (#10270-106, Gibco) were used to culture SW480 and DLD-1 cell lines, respectively. In addition, 10% (v/v) fetal bovine serum (FCS500, ExCell), 100 U/ml penicillin, and 100 μg/ml streptomycin (#15140-122, Gibco) were essential to Dulbecco’s modified Eagle’s medium and 1640. The cell lines were cultured at 37 °C in a humidified incubator with 5% CO_2_.

### Cell Migration Assay

For cell migration, 3 × 10^5^ cells in 200 μl of serum-free medium were placed in 8.0 mm, 24-well plate chamber inserts (354578, Corning Life Sciences) with medium containing 10% fetal bovine serum at the bottom of the inserts. After incubation for 24 h, the cells were fixed with 4% paraformaldehyde for 20 min and washed three times with PBS. Then, the cells were stained with 0.5% crystal violet blue for 30 min and washed five times with double-distilled water. A cotton swab was used to remove cells on the upper surface of the insert. Subsequently, images of the stained cells were captured by an OLYMPUS inverted microscope.

### Wound Healing Assay

The monolayer of cells on the 6-well plate was scraped in a straight line with a 10 μl pipette tip to form a wound. Detached cells were removed by PBS, and then cells were cultured in serum-free medium. The scratches were photographed with an OLYMPUS inverted microscope at 0 h and 48 h after injury. The gap width was analyzed using ImageJ software (https://imagej.nih.gov/ij/download.html), and the gap width at 0 h was normalized to 1. Each gap width was calculated among all the defined sites along the scratch. Data are presented as the mean of three independent experiments.

### Immunofluorescence

For immunofluorescence, 1 × 10^4^ cells in 100 μl of medium were placed in 96-well plates (6055300, PerkinElmer). Cells were fixed with 4% paraformaldehyde for 20 min, permeabilized, and blocked with 0.5% Triton X-100 (T8200, Solarbio) for 20 min. After incubation with Actin-Tracker Red-594 (C2205S, Beyotime) for 30 min, the cells were incubated further with 4′,6-diamidino-2-phenylindole (C0060, Solarbio) for 5 min at a 1:1000 dilution. Fluorescence images were captured using Opera Phenix Plus (PerkinElmer, HH14001000). Image analysis was performed with Harmony software (https://support.myharmony.com/en-cn/download).

## Results

### Multiomics Characterization of Colon Cancer in Eastern Asians

Seventy-six treatment-naive colon cancer patients without Lynch syndrome were recruited from the WCH (termed the WCH cohort) with informed consent (33, 34). Paired tumors (T) and DNTs were collected from these patients immediately after surgery and stored in liquid nitrogen until use. The clinical information of patients and the clinicopathological characteristics of tumors are summarized in supplemental Table S1. A schematic of the experimental design is shown in supplemental Fig. S1. WES identified 8833 mutated genes with 87,553 somatic mutations. TMT-based isobaric labeling was adopted for the relative quantification of proteins and phosphopeptides by MS. Common reference samples and continuous post-data acquisition quality controls for every 30 MS runs and 20 MS runs were applied for proteomic and phosphoproteomic analyses, respectively (Pearson’s correlation analysis, supplemental Figs. S2, *A* and *B* and S3, *A* and *B*). Assessments of quality control samples, inter-plex common references, and replicate sample reproducibility demonstrated the high quality and reproducibility of the data as well as the high stability of the data acquisition system (Pearson’s correlation analysis, supplemental Figs. S2, *A*–*D* and S3, *A*–*C*). The density distribution of adjusted intensity in the proteomics and phosphoproteomics also showed the quality of the samples and the MS data (supplemental Figs. S2*E* and S3*D*). Across the dataset, appropriate filtering resulted in the identification of 9281 proteins and 12,710 phosphorylation sites (localization probability >0.75) (supplemental Figs. S2*F* and S3*E*). Among all identified proteins, 5133 proteins quantified in more than 50% of both *APC*-MUT and *APC*-WT colon cancer cases with at least two identified unique peptides were used for subsequent data mining (supplemental Table S4). Overlapping analysis revealed that 831 phosphoproteins were identified in the proteome (supplemental Fig. S3, *F* and *G* and supplemental Table S4). Data imputation did not change the data distribution (supplemental Figs. S2*G* and S3*H*).

### Genomic Characteristics of *APC*-MUT Tumors

**Fig. 1:**
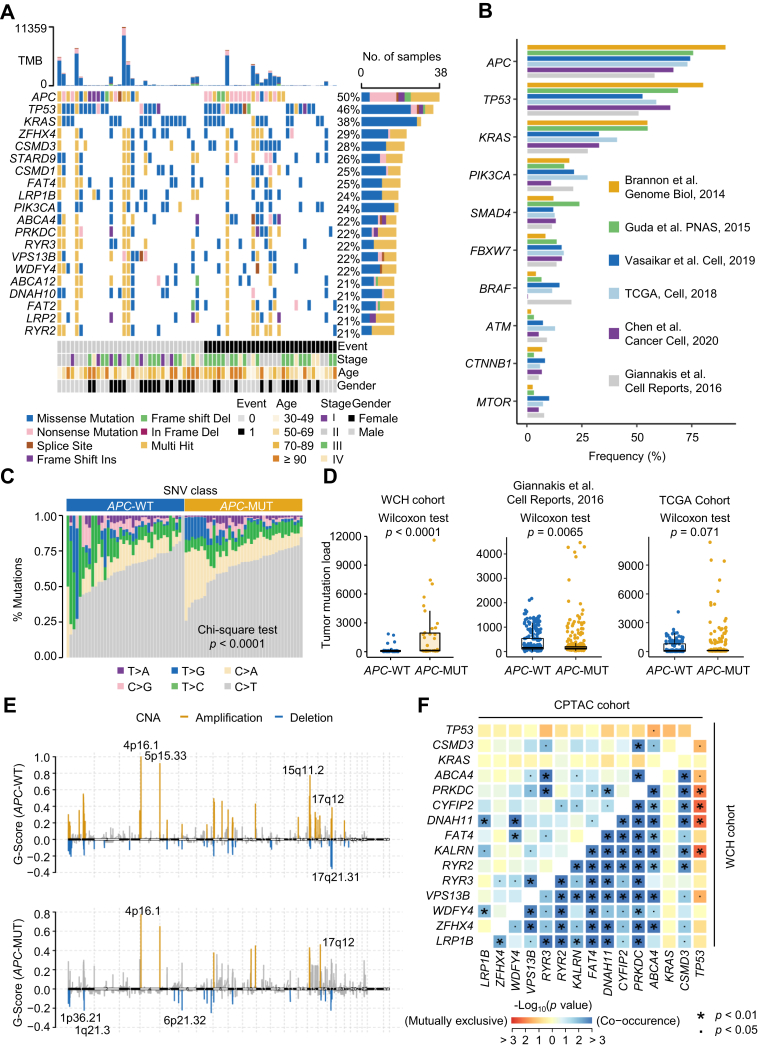
**Genomic comparisons between *APC*-MUT and *APC*-WT colon cancer.***A*, genetic profile and associated clinicopathologic features of all 76 colon cancer patients. The bar plot on the *top* indicates the total number of somatic mutations in each patient. The bar plot on the *right* represents the distribution and compositions of mutation types in each gene. *B*, bar plot of mutational frequency (%) for the top ten mutated genes in the previously published CRC databases. *C*, percentages of SNVs in *APC*-WT and *APC*-MUT tumors. Test methods, Chi-square test. *D*, comparison of tumor mutation load between *APC*-MUT and *APC*-WT tumors in the WCH cohort, Giannakis’ study, and TCGA cohort. Test methods, Wilcoxon rank-sum test. *E*, focal peaks with significant somatic copy number amplification (*yellow*) and deletion (*blue*) (GISTIC2 Q-values <0.1) shown in *APC*-MUT and *APC*-WT tumors, respectively. The top five amplified and deleted cytobands are labeled. *F*, the relationship between the somatic mutation frequencies of the first 15 mutated genes in *APC*-MUT tumors of the WCH cohort (*bottom right*), and the relationship of somatic mutation frequencies of the same 15 genes (*top left*) in *APC*-MUT tumors of the CPTAC cohort. Test methods, Fisher’s exact test. APC, Adenomatous polyposis coli; APC-MUT, APC-mutant; CRC, colorectal cancer; CPTAC, Clinical Proteomic Tumor Analysis Consortium; GISTIC, Genomic Identification of Significant Targets in Cancer; SNV, single nucleotide variant; TMB, Tumor mutation burden; WCH, West China Hospital.

WES showed that *APC* was the most frequently mutated gene in the WCH cohort, with a mutation frequency of up to 50% (Fig. 1*A* and supplemental Table S2), consistent with the findings of a total of 1562 CRC patients from six other public databases (Fig. 1*B* and supplemental Table S1) (20, 35, 36, 37, 38, 39, 40, 41, 42). By comparing the differences in genomic profiles between the *APC*-MUT and *APC*-WT subpopulations, we revealed that the proportion of total single nucleotide variants was significantly different (Chi-square test, *p* < 0.0001, Fig. 1*C*), and the conversion of cytosine to adenine (C>A) was more frequent in the *APC*-MUT tumors than in the *APC*-WT tumors. Statistical analysis of tumor mutational burden (TMB) showed that the TMB in the *APC*-MUT tumors was significantly higher than that in the *APC*-WT tumors (Wilcoxon rank-sum test, *p* < 0.0001, Fig. 1*D*). The results from two other cohorts also showed higher TMB in the *APC*-MUT tumors (Wilcoxon rank-sum test, *p* = 0.0065 and *p* = 0.071 for two other cohorts, Fig. 1*D*). Notably, a higher degree of dispersion of TMB values in the *APC*-MUT tumors was observed in all three cohort studies (Fig. 1*D*), suggesting a higher degree of heterogeneity in *APC*-MUT tumors than in *APC*-WT tumors. In addition, we also analyzed the SCNAs of *APC*-MUT and *APC*-WT tumors in the WCH cohort. The analysis of focal SCNAs showed that the cytobands where the variant site was located had obvious amplified regions at 5p15.33 and 15q11.2 in *APC*-WT tumors and deleted regions at 17q21.31 in *APC*-WT tumors and at 1p36.21, 1q21.3, and 6p21.32 in *APC*-MUT tumors (GISTIC2 Q-values <0.1, Fig. 1*E*). Association analysis of mutated genes in the *APC*-MUT tumors using the somatic interactions algorithm was performed. We found that mutations in *TP53* and *KRAS* were mutually exclusive with mutations in other major genes in *APC*-MUT tumors (Fisher’s exact test, *p* < 0.05, Fig. 1*F*), further suggesting high heterogeneity in *APC*-MUT tumors. Collectively, these results indicate the mutational differences between *APC*-MUT and *APC*-WT subtypes and the mutational heterogeneity of *APC*-MUT tumors.

### Proteomics and Phosphoproteomics Reveal the Heterogeneity of *APC*-MUT Colon Cancer

**Fig. 2:**
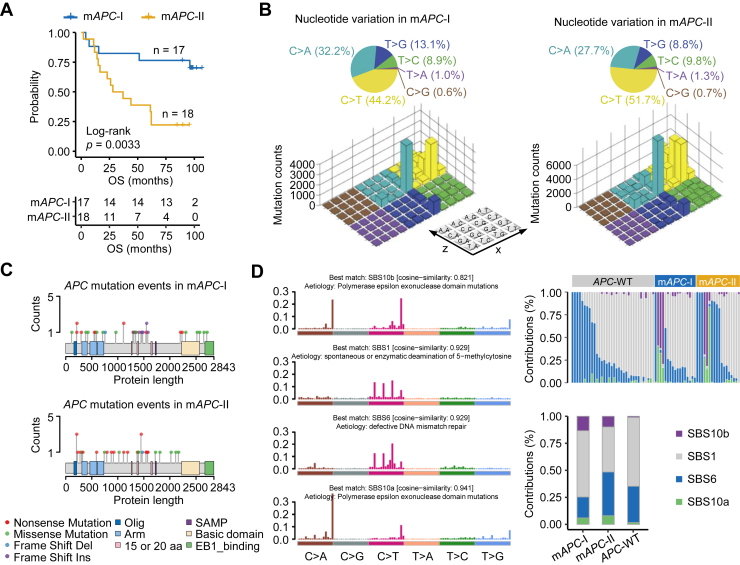
**Molecular classification of *APC*-MUT colon cancer and the genomic features of each subtype.***A*, Kaplan–Meier curves for colon cancer patients in the m*APC*-I and m*APC*-II subtypes from the *APC*-MUT subpopulation of the WCH cohort. Test methods, log-rank test. *B*, Lego plot representation of 96 nucleotide mutation patterns in colon cancer tumors. The 96 nucleotide mutation patterns are derived from six single-nucleotide substitutions (C>A, C>G, C>T, T>A, T>C, T>G) and their sequence contexts (the preceding and the following base). Each cell in the plane of x and z axes represents one kind of single-nucleotide substitution with its preceding and following bases. The y axis represents the mutation counts. The pie chart on the *top* shows the proportion of six major categories of nucleotide variation. The color of the pie charts and Lego plots represents one kind of single-nucleotide substitution. *C*, lollipop plot showing the types of *APC* mutations in the m*APC*-I and m*APC*-II subtypes. *D*, mutational activities of corresponding extracted mutational signatures, including SBS10b, SBS1, SBS6, and SBS10a (*left*). Contribution of mutational counts that attributed to corresponding mutational signatures in *APC*-MUT tumors and their subtypes (*top right*). Bar plots of contributions for the four mutational signatures in m*APC*-I, m*APC*-II, and *APC*-WT subpopulations (*bottom right*). APC, Adenomatous polyposis coli; APC-MUT, APC-mutant; APC-WT, APC-wildtype; WCH, West China Hospital.

To further explore the *APC*-MUT heterogeneity, we screened proteins and phosphosites whose intensities adjusted to the intensities in DNT were significantly correlated with OS (log-rank test, *p* < 0.05, supplemental Fig. S4, *A* and *B*) and performed unsupervised consensus clustering of *APC*-MUT patients using these proteins and phosphosites. As a result, the *APC*-MUT patients were divided into m*APC*-I and m*APC*-II subtypes, of which 17 were in m*APC*-I and 18 were in m*APC*-II (supplemental Fig. S4*C*). The plot of the silhouette width of unsupervised clustering showed that the *APC*-MUT colon cancer patients were accurately classified into different subtypes by integrative proteomics and phosphoproteomics (supplemental Fig. S4*D*). The CPTAC data were used as a validation set for the WCH data. The patients in CPTAC are mainly Westerners or persons lived in Western countries (supplemental Table S1). The results from the CPTAC cohorts also demonstrated that the *APC*-MUT patients were divided into CPTAC-m*APC*-I and CPTAC-m*APC*-II subtypes, of which 37 were in CPTAC-m*APC*-I and 38 were in CPTAC-m*APC*-II (log-rank test, *p* = 0.029, supplemental Fig. S4, *E*–*G*). Survival analysis showed a significant prognostic difference between the two subtypes, with patients in m*APC*-II having worse prognosis (log-rank test, *p* = 0.0033, Fig. 2*A* and supplemental Table S3). Subsequently, we analyzed 96 single base substitution types in the trinucleotide context in both the m*APC*-I and m*APC*-II subtypes of the WCH cohort. Pie charts showed that compared with the m*APC*-I subtype, the C > T transition in m*APC*-II was slightly increased (Fig. 2*B*). The Lego map showed that the C>A transition at the CpCpT site and the C>T transition at the ApCpG, CpCpG, and GpCpG sites were particularly prominent in the m*APC*-II subtype, indicating the importance of specific single base substitutions in *APC* mutational heterogeneity (Fig. 2*B*).

The oligomerization domain, armadillo repeats, 15 aa repeats, 20 aa repeats, and SAMP repeats are the main functional domains of *APC* (3). Next, we counted the mutation types in these domains of *APC* in m*APC*-I and m*APC*-II tumors. Our results showed that nonsense mutations were the main type of mutation in m*APC*-II, while m*APC*-I accounted for the largest proportion of missense mutations (Fig. 2*C*). Nonsense mutations, frameshift deletions, and frameshift insertions all lead to the production of *APC* truncations, which often have functions in promoting cell proliferation and cell migration (10). We next summarized four mutational signatures of the WCH cohort, including polymerase epsilon exonuclease domain mutations (SBS10a and SBS10b), spontaneous or enzymatic deamination of 5-methylcytosine (SBS1), defective DNA mismatch repair (MMR) (SBS6). We found that the proportion of SBS6 in m*APC*-II was significantly higher than that in m*APC*-I. Consistently, the total number of gene mutations' contribution in m*APC*-II was greater than that in m*APC*-I (Fig. 2*D*).

**Fig. 3:**
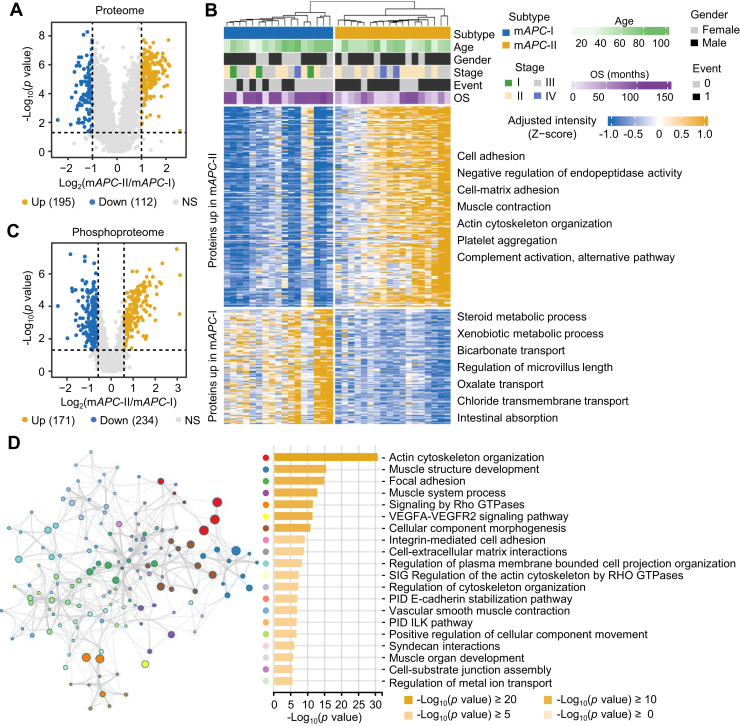
**Proteome and phosphoproteome signatures of *APC*-MUT subtypes.***A*, volcano plot showing 195 upregulated and 112 downregulated proteins in the m*APC*-II subtype of *APC*-MUT subpopulations from the WCH cohort. Test method, Wilcoxon rank-sum test. Cutoff, ratio (m*APC*-II/m*APC*-I) >2 or <0.5. *Blue* and *yellow dots* represent significantly differential proteins in m*APC*-I and m*APC*-II subtypes, respectively. *B*, enrichment analysis of differentially expressed proteins between the m*APC*-I and m*APC*-II subtypes of *APC*-MUT subpopulations from the WCH cohort. The subtype, age, gender, TNM stage, survival event, and OS of patients were annotated above the heatmap. The heatmap depicts the adjusted intensity of proteins with log_2_-transformation. The biological functions of these proteins are shown at the *right* of the heatmap. *C*, volcano plot showing 171 upregulated and 234 downregulated phosphosites in the m*APC*-II subtype of *APC*-MUT subpopulations from the WCH cohort. Test method, Wilcoxon rank-sum test. Cutoff, ratio (m*APC*-II/m*APC*-I) >1.5 or <0.667. *Blue* and *yellow dots* represent significantly differential phosphosites between the m*APC*-I and m*APC*-II subtypes, respectively. *D*, pathway enrichment analysis of the corresponding proteins of upregulated phosphosites in the m*APC*-II subtype using Metascape. The color of the nodes in the network represents different biological pathway terms. Node size is proportional to the number of input genes that are classified within that term, while node color represents the identity of the cluster to which it belongs. The edges linking terms with a similarity score greater than 0.3 are displayed, with the thickness of the edges representing the degree of similarity between biological pathways. APC, Adenomatous polyposis coli; APC-MUT, APC-mutant; OS, overall survival; WCH, West China Hospital.

To clarify the underlying reasons for the distinct clinical outcomes between m*APC*-I and m*APC*-II, we screened the proteins with expression differences between them. As a result, 195 upregulated and 112 downregulated proteins in m*APC*-II were identified (Wilcoxon rank-sum test, *p* < 0.05, ratio (m*APC*-II/m*APC*-I) >2 or <0.5, Fig. 3*A* and supplemental Table S4). Pathway enrichment showed enhanced metastasis-related pathways, such as cell adhesion, cell-matrix adhesion, and actin cytoskeleton organization in the m*APC*-II subtype, consistent with unfavorable clinical outcomes of patients in m*APC*-II (Wilcoxon rank-sum test, *p* < 0.05, ratio (m*APC*-II/m*APC*-I) >2 or <0.5, Fig. 3*B* and supplemental Table S4). In addition, we also obtained 171 upregulated and 234 downregulated phosphosites in m*APC*-II (Wilcoxon rank-sum test, *p* < 0.05, ratio (m*APC*-II/m*APC*-I) >1.5 or <0.667, Fig. 3*C* and supplemental Table S4). Pathway enrichment using their corresponding phosphoproteins showed that pathways such as actin cytoskeleton organization, focal adhesion, integrin-mediated cell adhesion, and cell–extracellular matrix interactions were significantly enriched in m*APC*-II, again indicating that tumor metastasis-related pathways were enriched in m*APC*-II (Fig. 3*D* and supplemental Fig. S5).

We further screened differential proteins between CPTAC-m*APC*-I and CPTAC-m*APC*-II, and 641 significantly upregulated and 65 significantly downregulated proteins in CPTAC-m*APC*-II were obtained (Wilcoxon rank-sum test, *p* < 0.05, ratio (CPTAC-m*APC*-II/CPTAC-m*APC*-I) >1.5 or <0.667, supplemental Fig. S6*A* and supplemental Table S8). Consistent with the enrichment results in the WCH cohort, metastasis-related pathways such as cell adhesion, cell-matrix adhesion, and extracellular matrix organization were also enriched in the CPTAC-m*APC*-II subtype (supplemental Fig. S6*B*). Overlapping analysis showed that 174 proteins were upregulated in both the WCH and CPTAC cohorts, and 61 of them were known targets of drugs approved or in clinical trials (supplemental Fig. S6*C*). The expression differences of the 61 proteins between m*APC*-I and m*APC*-II in the WCH and CPTAC cohorts were presented in supplemental Fig. S6*D* (Wilcoxon rank-sum test, *p* < 0.05, supplemental Table S8). Among the 61 proteins, 17 proteins, such as FLNA, FN1, COL1A1, ITGA5, and ITGB1, were closely related to metastasis with approved drugs or drugs in clinical trials (supplemental Fig. S6*E*). The results indicate that the 17 proteins are potential drug targets for the treatment of m*APC*-II colon cancer patients.

### RAI14 is a Key Prognostic Determinant for *APC*-MUT Colon Cancer Patients

**Fig. 4:**
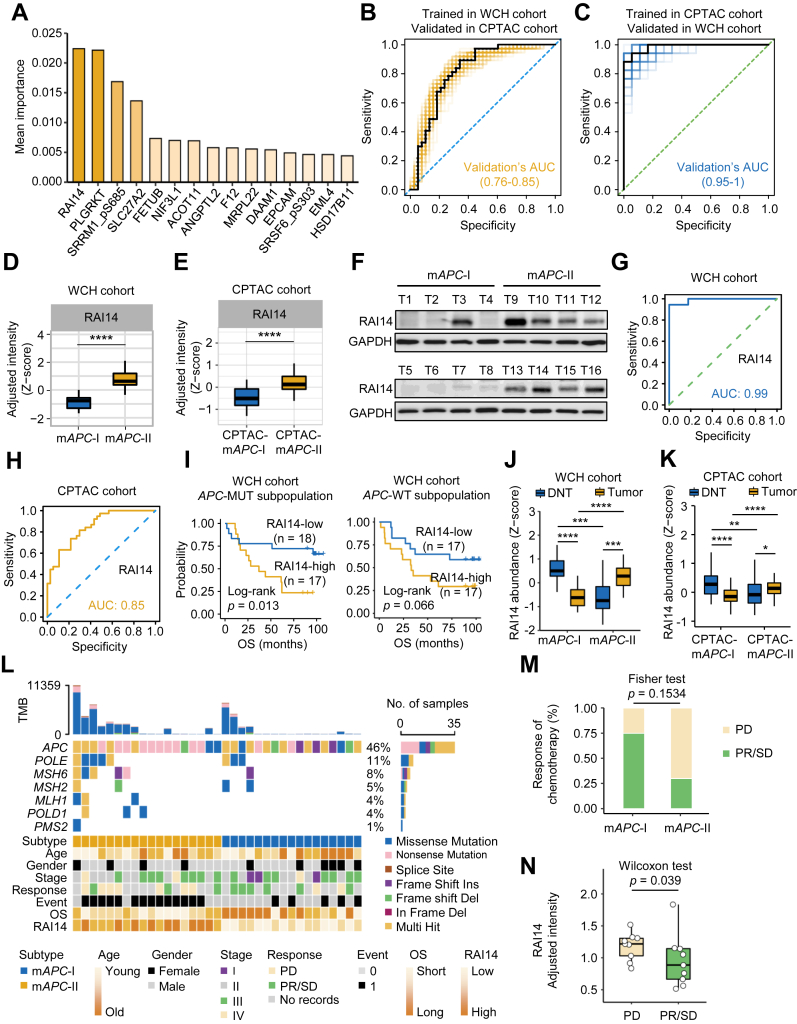
**Identification of RAI14 as a prognostic determinant from *APC*-MUT subpopulation.***A*, the most discriminative protein and phosphopeptide signatures selected by random forest. The 15 signature items included 13 proteins and two phosphopeptides. *B*, ROC curves for predicting m*APC*-I and m*APC*-II subtypes in CPTAC cohort with 101 10-fold cross validation RF-based models trained in WCH cohort. Curves were obtained by calculating the sensitivity and specificity of the assay at each possible cutoff point. The *yellow line* represents the ROC curves predicted using the signature molecules, except for two molecules with more than 50% missing values in the CPTAC cohort. The *black line* represents the ROC curve with median AUC. *C*, ROC curves for predicting CPTAC-m*APC*-I and CPTAC-m*APC*-II subtypes in WCH cohort with 101 10-fold cross validation RF-based models trained in CPTAC cohort. Curves were obtained by calculating the sensitivity and specificity of the assay at each possible cutoff point. The ROC curve for predicting the mAPC-I and mAPC-II subtypes using the signature molecules from (*A*) is shown in *blue*, except for two molecules with more than 50% missing values in the CPTAC cohort. The *black line* represents the ROC curve with the median AUC. *D* and *E*, the statistics of the relative protein expression of RAI14 in the m*APC*-I and m*APC*-II subtypes in the WCH cohort (*D*) and CPTAC cohort (*E*). Test method, Wilcoxon rank-sum test, ∗∗∗∗*p* < 0.0001, ∗∗∗*p* < 0.001, ∗∗ *p* < 0.01, ∗ *p* < 0.05, for indicated comparisons. *F*, immunoblots showed the expression of RAI14 in the m*APC*-I and m*APC*-II colon cancer tumors. *G*, ROC curves for predicting m*APC*-I and m*APC*-II subtypes using RAI14. Curves were obtained by calculating the sensitivity and specificity of the assay at each possible cutoff point. The *blue line* represents the ROC curve predicted only using RAI14. *H*, ROC curves for predicting CPTAC-m*APC*-I and CPTAC-m*APC*-II subtypes using RAI14. Curves were obtained by calculating the sensitivity and specificity of the assay at each possible cutoff point. The *yellow line* represents the ROC curve predicted only using RAI14. *I*, Kaplan–Meier curves for the *APC*-MUT subpopulation with high and low level of RAI14 in the WCH cohort and CPTAC cohort. Test method, log-rank test. *J* and *K*, the statistics of the relative protein expression of RAI14 in distant normal and tumorous tissues in the m*APC*-I and m*APC*-II subtypes of the WCH cohort (*J*) and CPTAC cohort (*K*). Test method, Wilcoxon rank-sum test, ∗∗∗∗*p* < 0.0001, ∗∗∗*p* < 0.001, ∗∗ *p* < 0.01, ∗ *p* < 0.05, for indicated comparisons. *L*, mutations of *APC* and MMR genes, as well as the corresponding chemotherapy response and survival events in m*APC*-I and m*APC*-II subtypes. The bar plot on the *top* indicates the total number of somatic mutations in each patient. The bar plot on the *right* represents the distribution and compositions of mutation types in each gene. *M*, statistics analysis of the chemotherapy response of patients in the m*APC*-I and m*APC*-II subtypes (Fisher's exact test). *N*, statistics of RAI14-adjusted intensity in colon cancer patients with PD and PR/SD (Wilcoxon rank-sum test). APC, Adenomatous polyposis coli; APC-MUT, APC-mutant; CPTAC, Clinical Proteomic Tumor Analysis Consortium; MMR, mismatch repair; PD, progressive disease; PR, partial remission; RF, random forest; SD, stable disease; WCH, West China Hospital.

To further identify the most discriminative characteristics between m*APC*-I and m*APC*-II, we calculated the contribution of proteins and phosphosites involved in subtyping using a RF learning model. As a result, there were 13 proteins and two phosphosites in the top 15 feature variables of contribution (Fig. 4*A* and supplemental Table S5). Interestingly, the top characteristic variables were able to well predict the m*APC*-I and m*APC*-II subtypes by 10-fold cross validation RF-based models in both the WCH (Fig. 4*B*) and CPTAC cohorts (Fig. 4*C*). RAI14 had the highest contribution in distinguishing m*APC*-I and m*APC*-II (Fig. 4*A*). More importantly, the AUC values of RAI14 alone were 0.99 in the WCH cohort and 0.85 in the CPTAC cohort, suggesting that the expression of RAI14 can distinguish m*APC*-I and m*APC*-II subtypes as well (Fig. 4, *G* and *H*). Statistical analysis of the adjusted RAI14 expression in m*APC*-I and m*APC*-II tumors showed much higher adjusted expression of RAI14 in m*APC*-II in both the WCH and CPTAC cohorts (Wilcoxon rank-sum test, Fig. 4, *D* and *E*). Comparison between APC-MUT and APC-WT colon cancers suggested a nonspecific relationship between RAI14 expression and APC states (supplemental Fig. S7, *A*–*C*), However, RAI14 exhibited more notable differences between the two subtypes in APC-MUT colon cancers than in APC-WT colon cancers (supplemental Fig. S7, *A*–*C*). Survival analysis of RAI14 showed that high expression of RAI14 was significantly correlated with unfavorable prognosis of colon cancer patients in the *APC*-MUT tumors but less significant in the *APC*-WT tumors (log-rank test, *p* = 0.013 for *APC*-MUT tumors and *p* = 0.066 for *APC*-WT tumors, Fig. 4*I*). Unexpectedly, compared with RAI14 levels in the corresponding DNT, we found that the expression of RAI14 was lower in m*APC*-I tumors but higher in m*APC*-II tumors in both the WCH and CPTAC cohorts (Wilcoxon rank-sum test, Fig. 4, *J* and *K*), which was further confirmed by immunoblots (Fig. 4*F*).

We next examined the effect of RAI14 expression on the response to chemotherapy in WCH cohort. No significant difference was observed in age, gender, or TNM stage between the m*APC*-I and m*APC*-II subtypes of *APC*-MUT colon cancer patients (Fig. 4*L*). The treatment guidelines for CRC suggest that functional loss of MMR genes is a low-risk factor, and mild treatment strategies will be adopted (43). We found that patients with MMR deficiency in m*APC*-II had a worse prognosis than patients with MMR deficiency in m*APC*-I (Fig. 4*L*), suggesting that more attention should be given to the treatment of patients with MMR deficiency in m*APC*-II. Comparison of colon cancer patients' chemotherapy response showed that patients in m*APC*-II subtype were less responsive to chemotherapy than those in the m*APC*-I subtype (Fig. 4*M*). More importantly, we found that more patients with higher RAI14 expression suffered from progressive disease (Fig. 4*N*). Collectively, these results indicate that RAI14 is an important variable determining the prognosis of *APC*-MUT colon cancer patients.

### RAI14 Modulates Cell Adhesion–Related Phosphoproteome to Affect Cancer Cell Migration

**Fig. 5:**
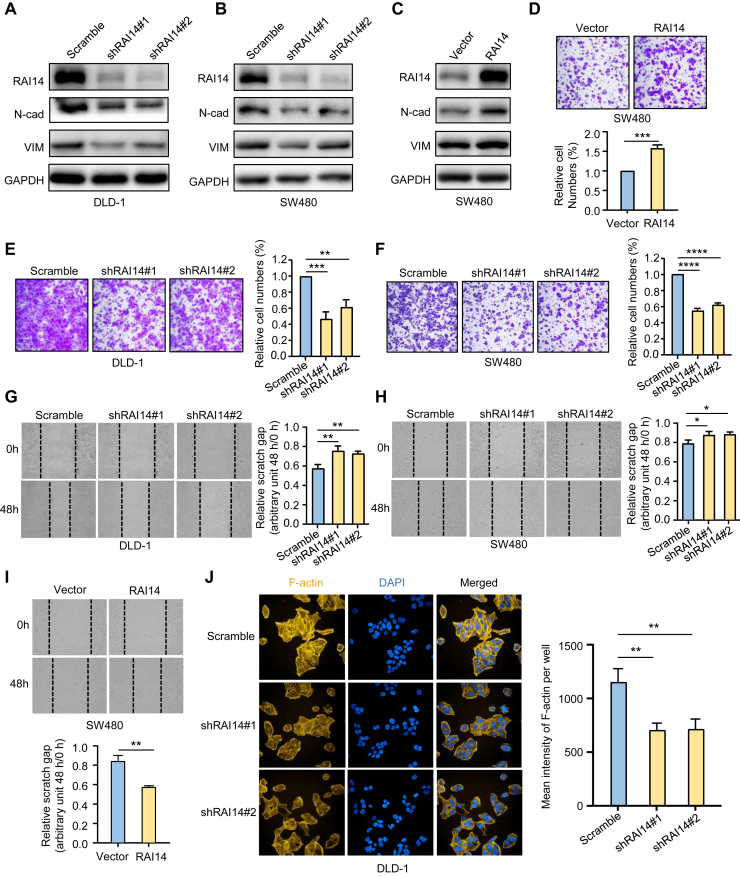
**High expression of RAI14 promotes colon cancer cell migration.***A*–*C*, Western blot analyses show the effects of RAI14 on the expression of EMT marker proteins in colon cancer cell lines. *D*–*F*, effects of RAI14 on cell migration evaluated by transwell assays in response to RAI14 knockdown (*E* and *F*) and overexpression (*D*). Test method, Student’s *t* test, ∗∗∗∗*p* < 0.0001, ∗∗∗*p* < 0.001, ∗∗ *p* < 0.01, ∗ *p* < 0.05, for indicated comparisons. *G*–*I*, wound healing assays show cell migration of colon cancer cells after 48 h in response to RAI14 knockdown (*G* and *H*) and overexpression (*I*). Test method, Student’s *t* test, ∗∗∗∗*p* < 0.0001, ∗∗∗*p* < 0.001, ∗∗*p* < 0.01, ∗*p* < 0.05, for indicated comparisons. *J*, effects of RAI14 silencing on the formation of F-actin determined by immunofluorescence assays. Test method, Student’s *t* test, ∗∗∗∗*p* < 0.0001, ∗∗∗*p* < 0.001, ∗∗*p* < 0.01, ∗*p* < 0.05, for indicated comparisons. EMT, epithelial-mesenchymal transition.

To reveal the roles of RAI14 in colon cancer, we knocked down RAI14 in the *APC*-mutated colon cancer cell lines DLD-1 and SW480 and overexpressed RAI14 in the SW480 cell line. Both transwell and scratch experiments showed that RAI14 knockdown dramatically inhibited cancer cell migration, while RAI14 overexpression reversed this phenotype (Fig. 5, *D*–*I*), consistent with the poor prognosis of colon cancer patients with high levels of RAI14 in tumors. A similar experiment was carried out in APC-WT colon cancer cell line RKO. The effect of RAI14 silence on RKO cell migration was weaker than that in *APC*-mutated colon cancer cell lines DLD-1 and SW480 (supplemental Fig. S7, *D* and *E*). Immunofluorescence experiments showed that after knockdown of RAI14, the production of F-actin was significantly inhibited, further demonstrating the role of RAI14 in regulating cell migration (Fig. 5*J*). Furthermore, immunoblotting showed that metastasis-related markers such as N-cadherin and vimentin were significantly decreased after RAI14 knockdown but dramatically upregulated in response to RAI14 overexpression (Fig. 5, *A*–*C*). Collectively, these results demonstrate that RAI14 is an important protein involved in the regulation of cancer cell migration.

**Fig. 6:**
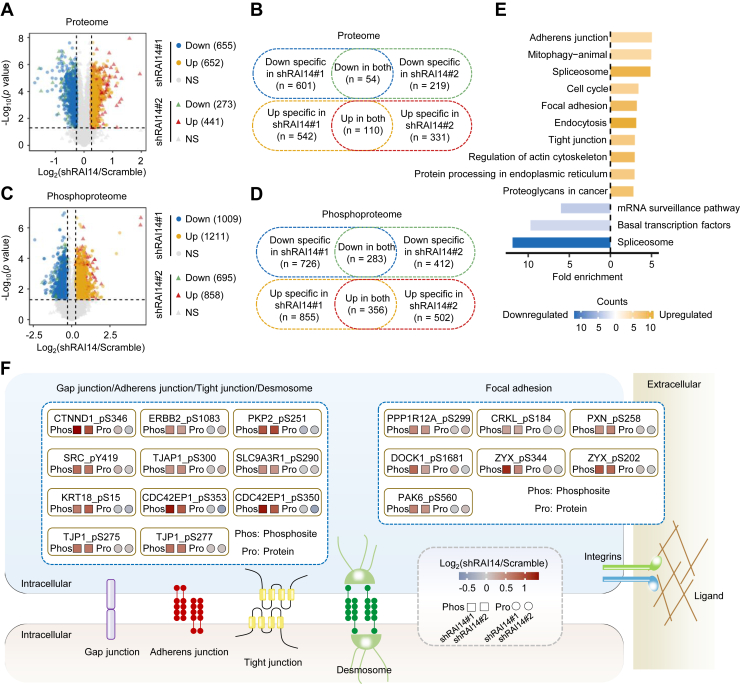
**RAI14-modulated phosphoproteome is associated with cell adhesion.***A*, volcano plot showing 655 downregulated and 652 upregulated proteins in RAI14 knockdown cells using the shRAI14#1 plasmid and 273 downregulated and 441 upregulated proteins in RAI14 knockdown cells using the shRAI14#2 plasmid. Test method, Student’s *t* test. Cutoff, ratio (shRAI14/Scramble) >1.2 or <0.833. Three biological repeats were performed for each RAI14 knockdown cell line. *B*, Venn diagram showing the number of overlapping proteins downregulated or upregulated in both RAI14 knockdown cell lines using shRAI14#1 and shRAI14#2 plasmids. *C*, volcano plot showing 1009 downregulated and 1211 upregulated phosphosites in RAI14 knockdown cells using the shRAI14#1 plasmid and 695 downregulated and 858 upregulated phosphosites in RAI14 knockdown cells using the shRAI14#2 plasmid. Test method, Student’s *t* test. Cutoff, ratio (shRAI14/Scramble) >1.2 or <0.833. Three biological repeats were performed for each RAI14 knockdown cell line. *D*, Venn diagram showing the number of overlapping phosphosites downregulated and upregulated in both RAI14 knockdown cell lines using the shRAI14#1 and shRAI14#2 plasmids. *E*, pathway enrichment analysis of the corresponding proteins of 283 downregulated and 356 upregulated phosphosites in both RAI14 knockdown cell lines using KEGG. *Yellow* represents upregulated pathways, and *blue* represents downregulated pathways. *F*, schematic of the cell adhesion pathway, showing the trends of phosphosites and the corresponding proteins involved in cell-cell adhesion and focal adhesion in response to RAI14 knockdown. Test method, Student’s *t* test. Cutoff, ratio (shRAI14/Scramble) >1.2.

To explore the underlying mechanisms by which RAI14 affects the metastasis of colon cancer cells, we analyzed the proteomic and phosphoproteomic changes in DLD-1 cells after RAI14 knockdown, including two shRNAs and three repeats per shRNA. As a result, a total of 9502 proteins and 14,098 phosphosites on 3299 proteins were identified (supplemental Fig. S8, *A*–*D*, and supplemental Table S8). Principal component analysis of the proteome and phosphoproteome data showed good separation of the control and RAI14-knockdown cells (supplemental Fig. S8, *E* and *F*). We next performed differential screening of the proteome and phosphoproteome. The results based on the proteome showed that 54 downregulated and 110 upregulated proteins were obtained in both RAI14 knockdown cell lines (Student’s *t* test, *p* < 0.05, ratio (shRAI14/Scramble) >1.2 or <0.833, Fig. 6, *A* and *B* and supplemental Table S6). Pathway analysis showed that the negative regulation of actin filament polymerization was significantly enriched, indicating that RAI14 is closely related to the depolymerization of F-actin (supplemental Fig. S8*G*), consistent with previous phenotypic observations (Fig. 5*J*). In addition, we identified 283 downregulated and 356 upregulated phosphosites after RAI14 knockdown (Student’s *t* test, *p* < 0.05, ratio (shRAI14/Scramble) >1.2 or <0.833, Fig. 6, *C* and *D* and supplemental Table S6). Pathway analysis of their corresponding phosphoproteins also showed that cell adhesion–related pathways, such as adherens junction, focal adhesion, tight junction, and regulation of actin cytoskeleton, were significantly enriched (Fig. 6*E*). Several cell adhesion–related phosphosites affected by RAI14 were presented (Fig. 6*F* and supplemental Table S6), suggesting that RAI14 might affect cell migration through modulating the cell adhesion–related phosphoproteome.

**Fig. 7:**
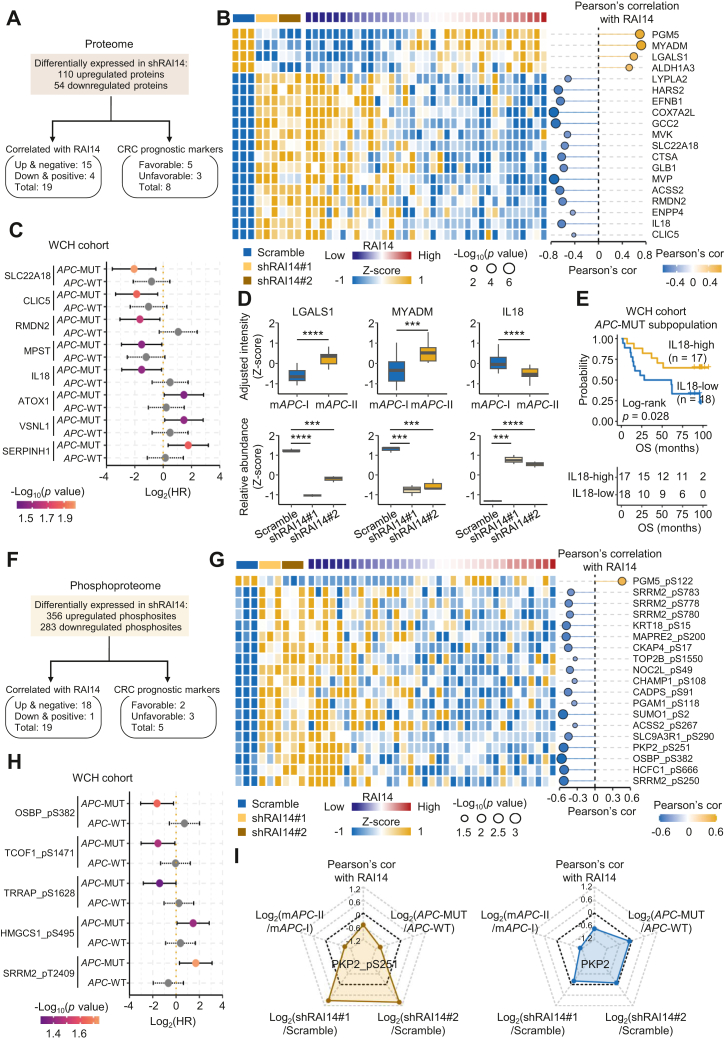
**Clinical implications of RAI14-dependent proteins and phosphosites.***A*, schematic of screening RAI14-dependent proteins based on the WCH cohort. The screening of differential proteins was carried out using Student’s *t* test (*p* < 0.05) and cutoff ratio (shRAI14/Scramble) >1.2 or <0.833. The screening of RAI14-correlated proteins in colon cancer tissues was performed using Pearson’s correlation analysis (*p* < 0.05). *B*, the expression of RAI14-dependent and correlated proteins. The cell lines and RAI14 levels were annotated above the heatmap. Heatmap depicts the relative expression of proteins with log_2_-transformation. The bubble chart describes the Pearson’s correlation coefficients of proteins correlated with RAI14. *C*, univariate Cox regression analysis of the RAI14-dependent proteins in *APC*-MUT and *APC*-WT subpopulations. Using Cox proportional hazards regression analysis, *p* < 0.05 was considered as significant. *D*, statistics of the relative expression of the cell adhesion proteins LGALS1, MYADM, and IL18 in the mAPC-I and mAPC-II subtypes and in response to RAI14 silencing. Test method for comparisons in colon cancer tissues, Wilcoxon rank-sum test, ∗∗∗∗*p* < 0.0001, ∗∗∗*p* < 0.001. Test method for comparisons in cell lines, Student’s *t* test, ∗∗∗∗*p* < 0.0001, ∗∗∗*p* < 0.001. *E*, Kaplan–Meier curves for *APC*-MUT subpopulations with high and low levels of IL18 in the WCH cohort (log-rank test). *F*, schematic of screening RAI14-dependent phosphosites based on the WCH cohort. The screening of differential phosphosites was performed using Student’s *t* test (*p* < 0.05) and cutoff ratio (shRAI14/Scramble) >1.2 or <0.833. The screening of RAI14-correlated phosphosites in colon cancer tissues was performed using Pearson’s correlation analysis (*p* < 0.05). *G*, the relative expression of RAI14-dependent phosphosites. The cell lines and RAI14 levels were annotated above the heatmap. Heatmap depicts the relative expression of phosphosites with log_2_-transformation. The bubble chart describes the Pearson’s correlation coefficients of phosphosites correlated with RAI14. *H*, univariate Cox regression analysis of the RAI14-dependent phosphosites in *APC*-MUT and *APC*-WT subpopulations, respectively, using Cox proportional hazards regression analysis, and *p* < 0.05 was considered as significant. *I*, radar charts include five types of information of PKP2_pS251 and PKP2, including the relative expression in two RAI14 knockdown cell lines (Log_2_(shRAI14/Scramble), Student’s *t* test), the relative expression between m*APC*-II and m*APC*-I subtypes (Log_2_(m*APC*-II/m*APC*-I), Wilcoxon rank-sum test), the relative expression between *APC*-MUT and *APC*-WT subpopulations (Log_2_(*APC*-MUT/*APC*-WT), Wilcoxon rank-sum test), and Pearson’s correlation with RAI14. APC, Adenomatous polyposis coli; APC-MUT, APC-mutant; WCH, West China Hospital.

To further explore the clinical relevance of the above RAI14-related phosphosites and proteins, correlation analysis based on the 110 upregulated and 54 downregulated proteins in both RAI14-knockdown cell lines was performed (Fig. 7*A*). As a result, we found that 15 increased proteins after knockdown of RAI14 were significantly negatively correlated with RAI14 in *APC*-MUT tumors, and four decreased proteins after RAI14 knockdown were significantly positively correlated with RAI14 in *APC*-MUT tumors (Pearson’s correlation analysis, *p* < 0.05, Fig. 7, *A* and *B* and supplemental Table S7). In addition, eight proteins were significantly correlated with the OS of *APC*-MUT colon cancer patients (Cox proportional hazards regression analysis, *p* < 0.05) but were not correlated with that of *APC*-WT colon cancer patients (Fig. 7, *A* and *C*, and supplemental Table S7). Notably, among these proteins, IL18, LGALS1, and MYADM are known to be involved in cell adhesion and cell migration (Wilcoxon rank-sum test in tissues and Student’s *t* test in cell line, Fig. 7*D*). Moreover, the high expression of IL18 in *APC*-MUT colon cancer patients predicted good prognosis in the WCH cohort (log-rank test, *p* = 0.028, Fig. 7*E*). We next performed a similar correlation analysis based on 356 upregulated and 283 downregulated phosphosites (Fig. 7*F*). The results showed that 18 increased phosphosites after RAI14 knockdown were significantly negatively correlated with RAI14 in *APC*-MUT tumors, and one reduced phosphosite after RAI14 knockdown was significantly positively correlated with RAI14 in *APC*-MUT tumors (Pearson’s correlation analysis, *p* < 0.05, Fig. 7, *F* and *G* and supplemental Table S7). In addition, five phosphosites were significantly correlated with the OS of *APC*-MUT colon cancer patients (Cox proportional hazards regression analysis, *p* < 0.05) but were not correlated with that of *APC*-WT colon cancer patients (Fig. 7*H* and supplemental Table S7). PKP2 is protein in cell adhesion pathway. After RAI14 knockdown in the cell line, the phosphorylation of PKP2 at S251 (PKP2_pS251) but not PKP2 itself was obviously increased. Interestingly, PKP2_pS251 was significantly higher in m*APC*-I than in m*APC*-II, suggesting PKP2_pS251 as a potential downstream effector of RAI14 affecting cell migration (Fig. 7*I*). Further investigation of the role of PKP2_pS251 is ongoing.

## Discussion

In this study, we performed an integrated analysis of genome, proteome, and phosphoproteome data of colon cancer, disclosed the phenotypic heterogeneity of *APC*-MUT tumors, and identified prognostic biomarkers for *APC*-MUT colon cancer patients. In *APC*-MUT colon cancer patients with poor prognosis, tumor metastasis-related processes and signaling pathways, such as cell adhesion, cell-matrix adhesion, actin cytoskeleton organization, and complement activation, were activated. Of note, RAI14 was identified as a key prognostic indicator for *APC*-MUT colon cancer in both East Asians and Westerners, and high levels of RAI14 were linked to unfavorable prognosis. Moreover, we found that knockdown of RAI14 led to reduced cell migration and changes in some epithelial-mesenchymal transition (EMT)-related markers. Mechanistically, knockdown of RAI14 was able to remodel the phosphoproteome associated with cell adhesion, which might alter the expression of certain EMT markers and promote F-actin degradation. Given that more than 50% of CRC patients have *APC* mutations, the prognostic utility of RAI14 in *APC*-MUT colon cancer may provide early warning and increase the chance of successful treatment.

*APC* mutation is considered to be one of the initiating factors of CRC (44). However, malignant progression of CRC may be directly, indirectly, or even unrelated to *APC* mutations (3). Therefore, prognosis prediction in patients with *APC*-MUT colon cancer is very difficult. On the one hand, *APC* mutations produce multiple stable APC truncations that affect WNT–β-catenin pathway activation. APC truncations may also acquire new functions with novel implications for tumor progression (3). On the other hand, in addition to APC mutations, tumors also develop other genetic mutations, such as *KRAS* and *TP53* mutations commonly found in CRC. These mutations may activate other signaling pathways and increase tumor malignancy (13). Molecular subtyping based on proteomic and phosphoproteomic data ignores the influence of genetic background, simplifies tumor heterogeneity characterization, and provides potential biomarkers and therapeutic targets for each subtype with *APC* mutations (16, 17, 18, 19). Molecular subtyping of *APC*-MUT colon cancer identifies a combination of molecular markers predicting prognosis in *APC*-MUT tumors, in which high expression of RAI14 in tumors is associated with poor prognosis in both WCH and CPTAC cohorts. Interestingly, the mutation frequencies of a number of driver genes such as *KRAS* and *TP53* were not significantly different between the two subtypes (supplemental Fig. S4, *H* and *I*), suggesting that proteomics-based prognosis prediction is more accurate.

Various molecular subtyping systems have been developed for CRC. In our study, we investigated the association of APC-mutated subtypes with reported molecular subtypes by comparing them with consensus molecular subtypes (CMSs) (45), TCGA mRNA subtypes (46), and CPTAC proteomic subtypes (19). Remarkably, we found that the mAPC-II subtype closely resembled the CMS4 subtype, as both subtypes showed worse OS and upregulation of genes linked to EMT (45). Additionally, the mAPC-II subtype was similar to subtype C of the CPTAC study, which was also associated with poor prognosis. The genes in the up-signature for subtype C were significantly enriched with collagens and extracellular matrix organization markers of EMT, thus strengthening the association of subtype C with poor prognosis and linking it to EMT activation (19). Notably, subtype C showed significant overlap with the TCGA MSI/CIMP subtype. Taken together, our findings suggest that the mAPC-II subtype, CMS4, and CPTAC subtype C with poor prognosis are all associated with EMT activation.

We find, for the first time, that RAI14 plays a decisive role in the prognosis of *APC*-MUT colon cancer. RAI14 consists of six ankyrin repeats in the N-terminal region and a long coiled-coil domain in the C-terminal region. Although RAI14 contains a putative nuclear localization signal peptide (P^270^KKRKAP^276^) (47), this signal may be masked by an intramolecular sequence or by interacting molecules, making full-length RAI14 inaccessible to nuclei. Notably, RAI14 can be induced by retinoic acid (RA) during development (48) and plays a vital role in the early morphogenesis of neurons (49). ALDH is a key component of RA signaling, and inactivation of *APC*s in familial adenomatous polyposis patients delays the maturation of colonic ALDH+ stem cells, suggesting a link between WNT and RA signaling (50). However, direct evidence of whether *APC* mutations can activate the RA signaling pathway is still lacking. Due to the strong expression correlation between RAI14 and enzymes associated with RA biosynthesis in colon cancer (supplemental Fig. S9, *A*–*H*), targeting the RA biosynthesis pathway to reduce the expression of RAI14 may be a promising therapeutic strategy for m*APC*-II colon cancer patients.

RAI14 acts as an actin-binding protein and is vital to maintain actin filament bundles. It can regulate F-actin dynamics at ectoplasmic specialization in rat testis (51, 52) and is dispensable for F-actin organization at the apical ectoplasmic specialization in mouse testis (53). A previous study showed that RAI14 is indirectly associated with actin cytoskeleton structures (54). In this study, we found that RAI14 silencing can remodel the phosphoproteome associated with cell adhesion, suggesting that RAI14 may regulate cell migration through changing the phosphoproteome. Notably, phosphorylation has been demonstrated to be an important regulator of cell adhesion molecules and F-actin, which affects cell migration (55). For example, integrin phosphorylation has proven to be of great importance, and pS759 and pS762 of β5-integrins are reported to promote cell migration (55). Phosphorylation of paxillin at S273 has also been demonstrated to be a key regulator of cell migration (56). In contrast, FLNA phosphorylation at S2152 weakens the ability of FLNA to bind to integrins (57). All this evidence implies RAI14 may affect cell migration through modulating cell adhesion–related phosphoproteome.

CRC single-cell transcriptome data show that in addition to being expressed in tumor cells, high levels of RAI14 are also observed in endothelial cells, fibroblasts, and myo-fibroblasts (58). Moreover, the expression of RAI14 in tumor cells is positively correlated with the number of fibroblasts and myo-fibroblasts. Fibroblasts are involved in the modulation of many components of the immune system, potentially leading to tumor cell immune evasion and reducing the effectiveness of cancer immunotherapy (59). In addition, RAI14 expression in gastric cancer is also found to be positively correlated with the infiltration levels of immune cells and OS (60). The above evidence suggests that the unfavorable prognosis associated with high expression of RAI14 in *APC*-MUT colon cancer may also be attributed to its immunoregulatory functions.

## Data Availability

All mass spectrometry raw data and output tables have been deposited to the ProteomeXchange Consortium and are available using the iProX accession: PXD038081, and the annotated spectra has been deposited to the MS-Viewer with the unique search key for each batch of proteomic or phosphoproteomic data (both tissue and cell samples) provided in the supplemental Table S4. The GSE raw sequence data reported in this paper have been deposited in the Genome Sequence Archive (61) in the National Genomics Data Center (62), China National Center for Bioinformation/Beijing Institute of Genomics, Chinese Academy of Sciences (GSA-Human: HRA003386), which are publicly accessible at https://ngdc.cncb.ac.cn/gsa-human.

## Supplemental data

This article contains supplemental data.

## Conflict of interest

There is no competing financial interest.

## Supplemental Data

Supplemental Figures

Supplemental Figures and Tables Caption
