# Supplementary material for: Phenotypic Heterogeneity Analysis of APC-Mutant Colon Cancer by Proteomics and Phosphoproteomics Identifies RAI14 as a Key Prognostic Determinant in East Asians and Westerners
Source: Mol Cell Proteomics. 2023 Mar 18;22(5):100532. doi: 10.1016/j.mcpro.2023.100532 (PMC10148045; doi:10.1016/j.mcpro.2023.100532)

supplemental Fig. S1

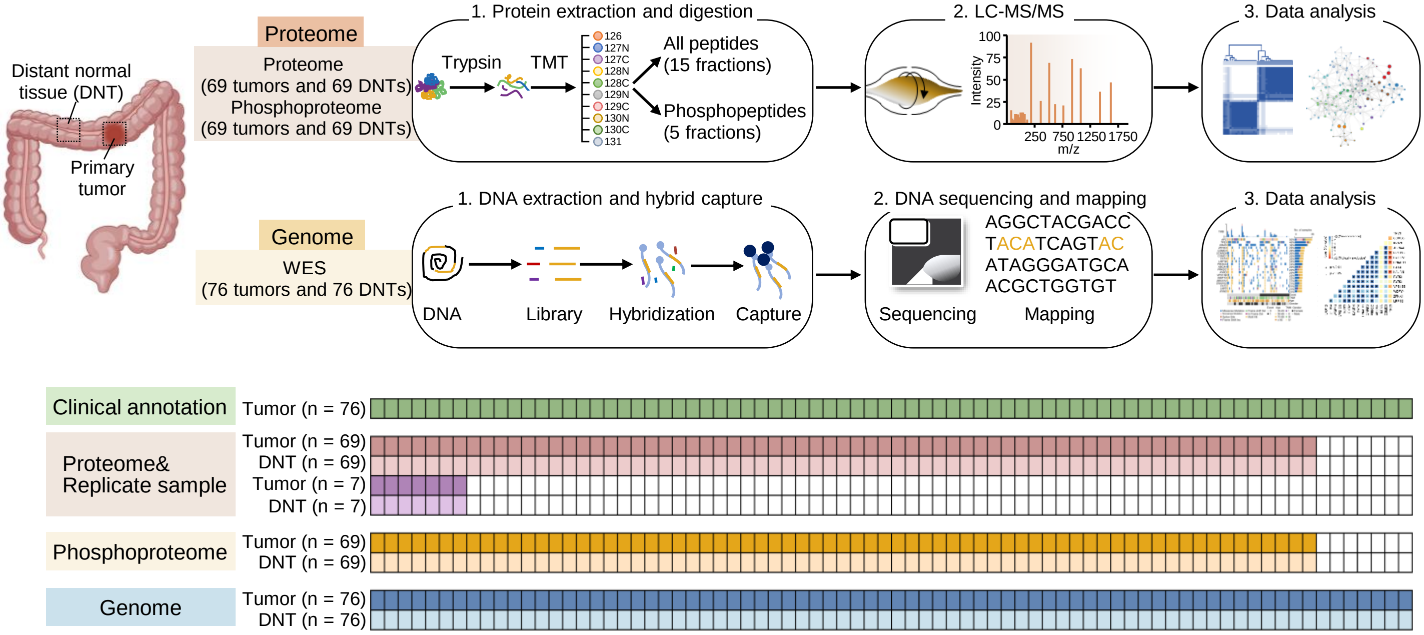

supplemental Fig. S2

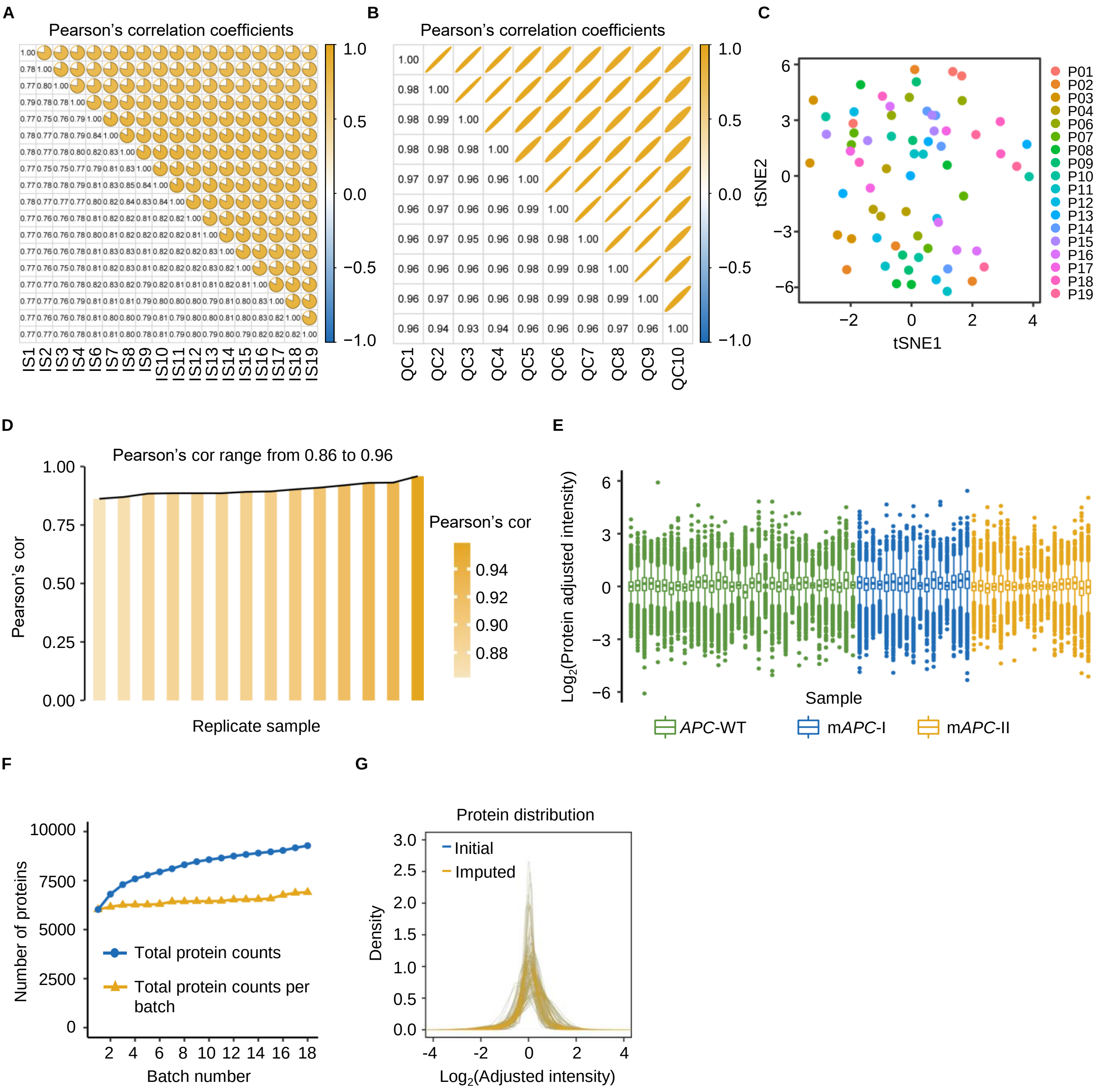

supplemental Fig. S3

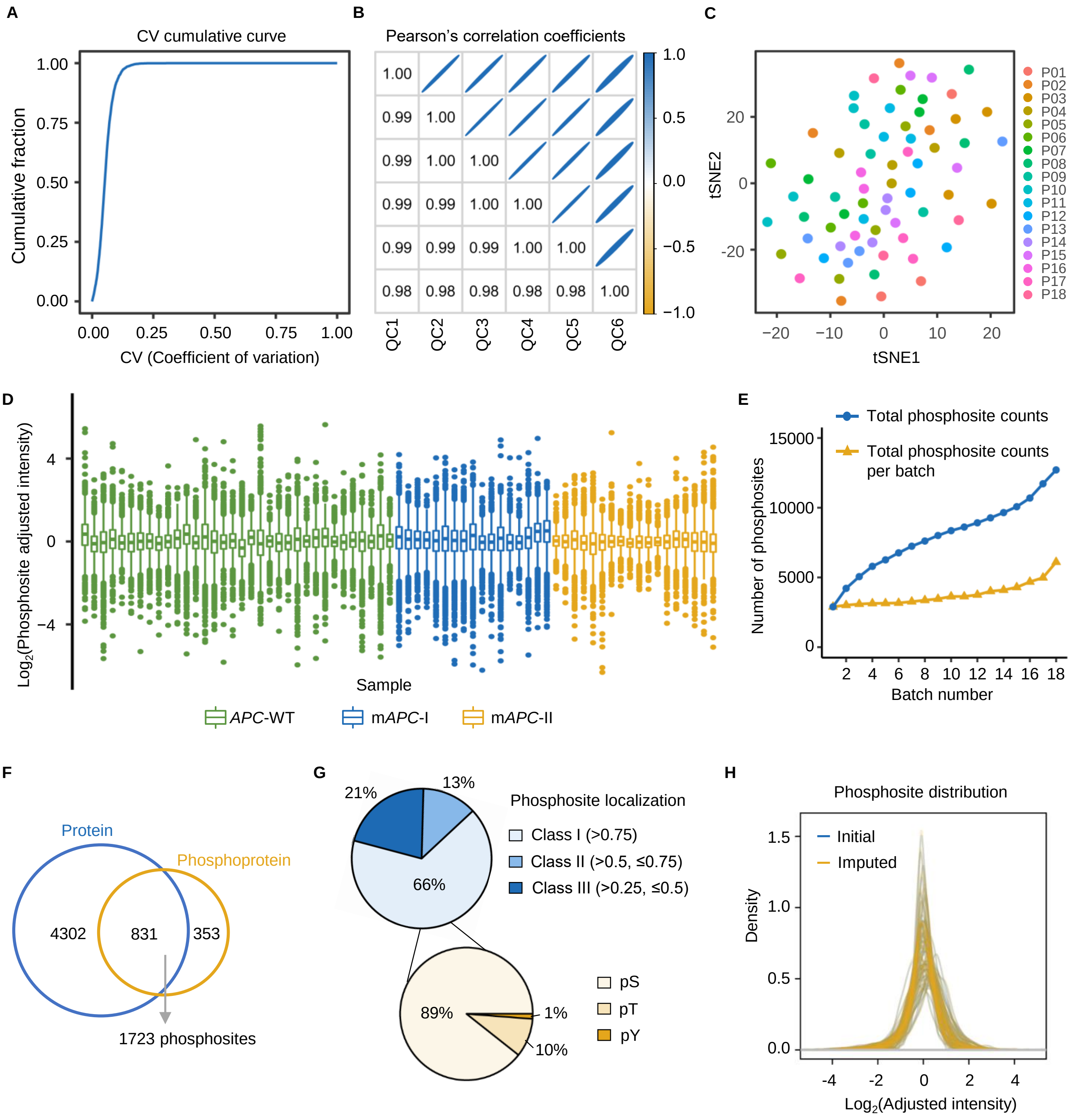

supplemental Fig. S4

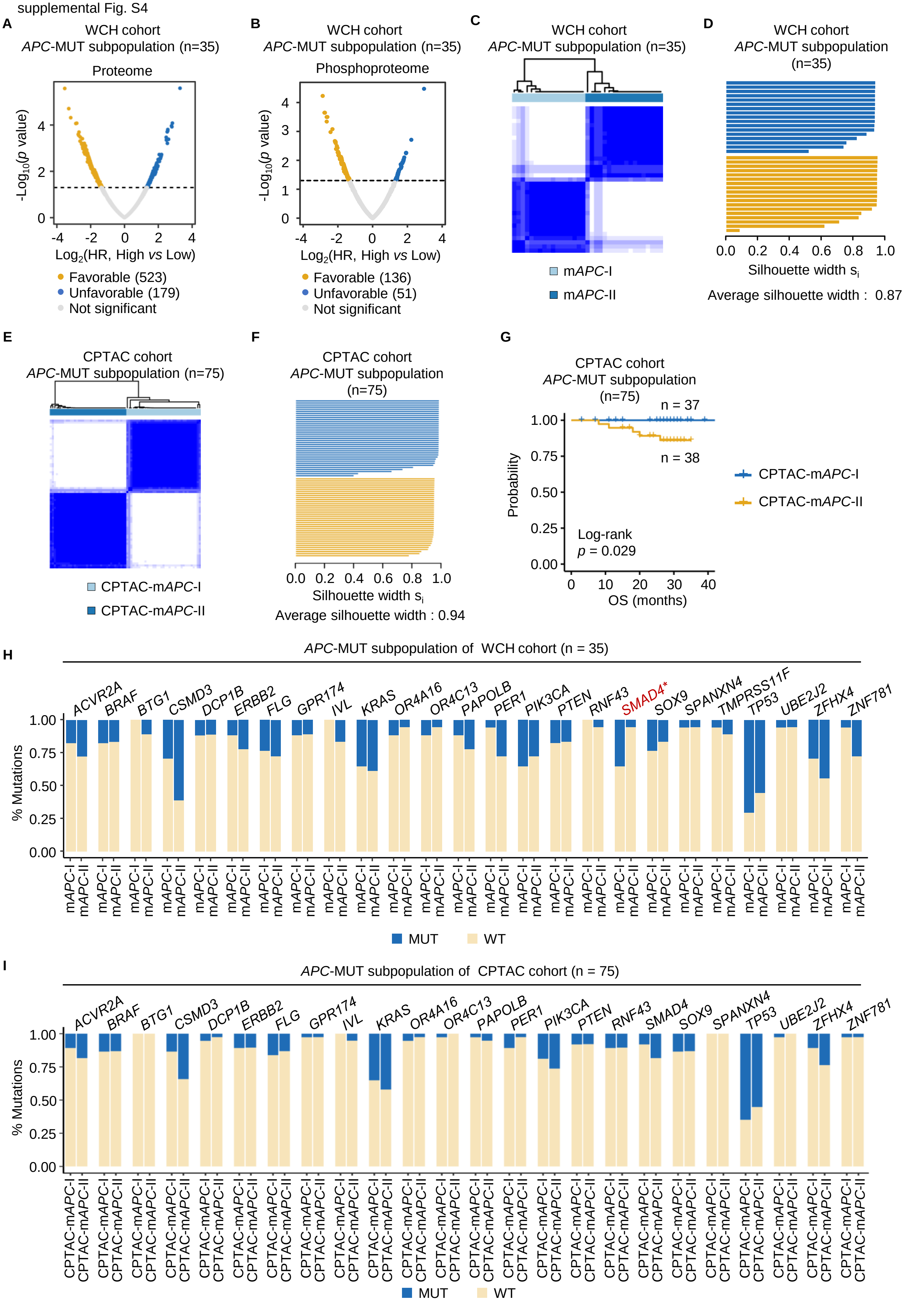

supplemental Fig. S5

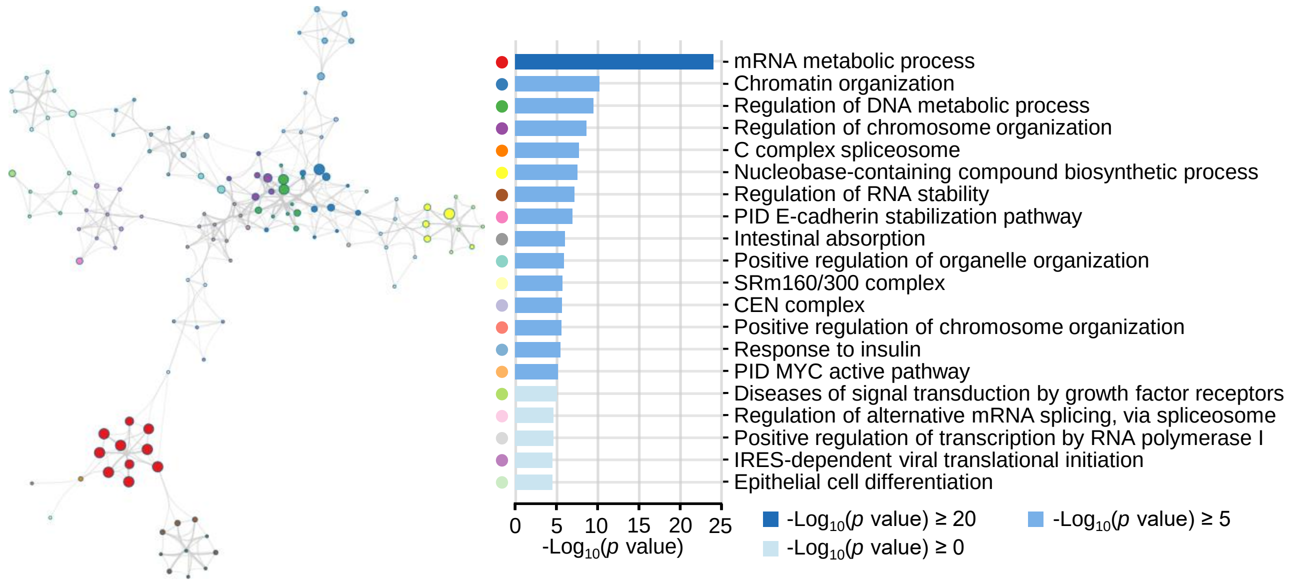

supplemental Fig. S6

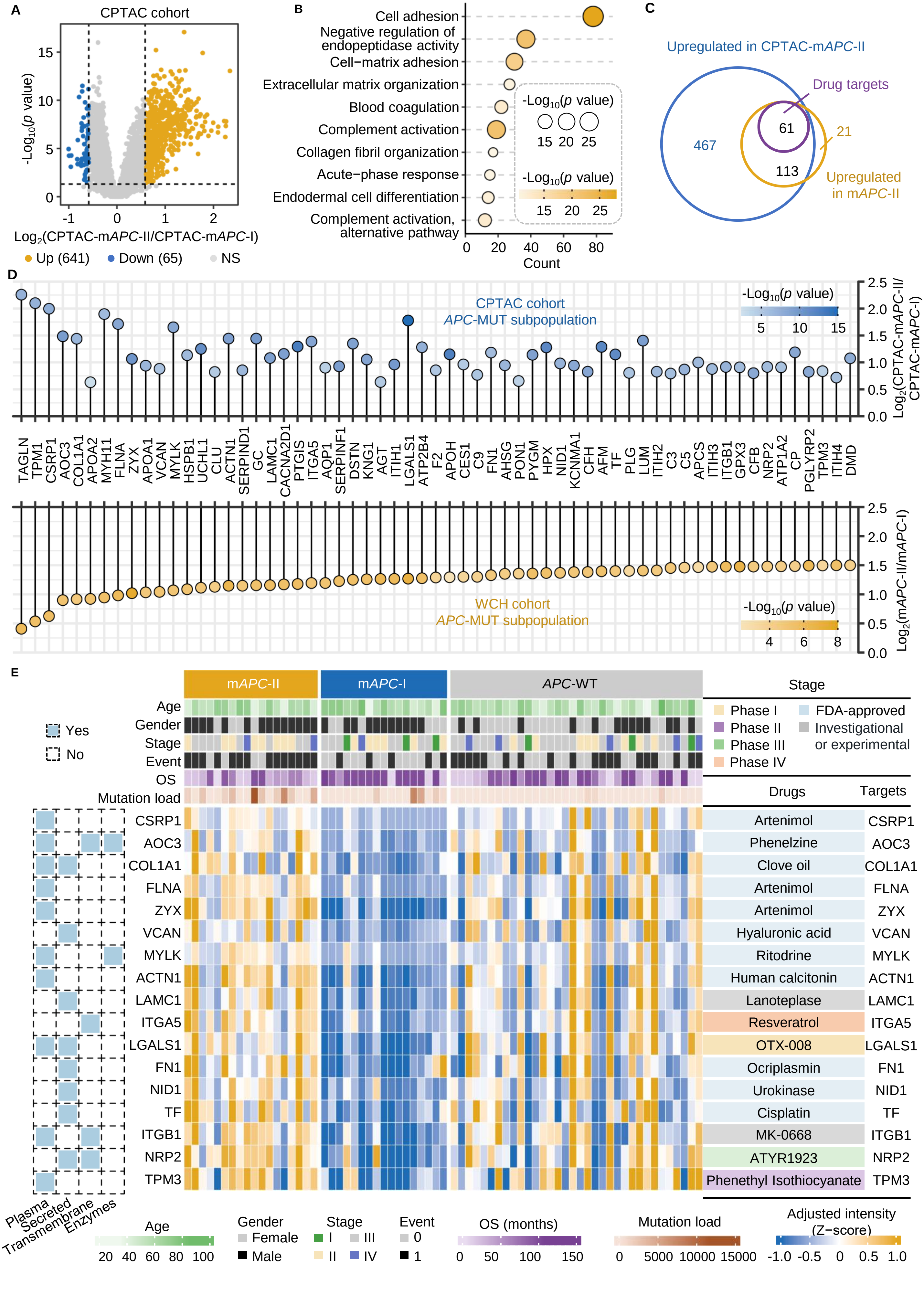

supplemental Fig. S7

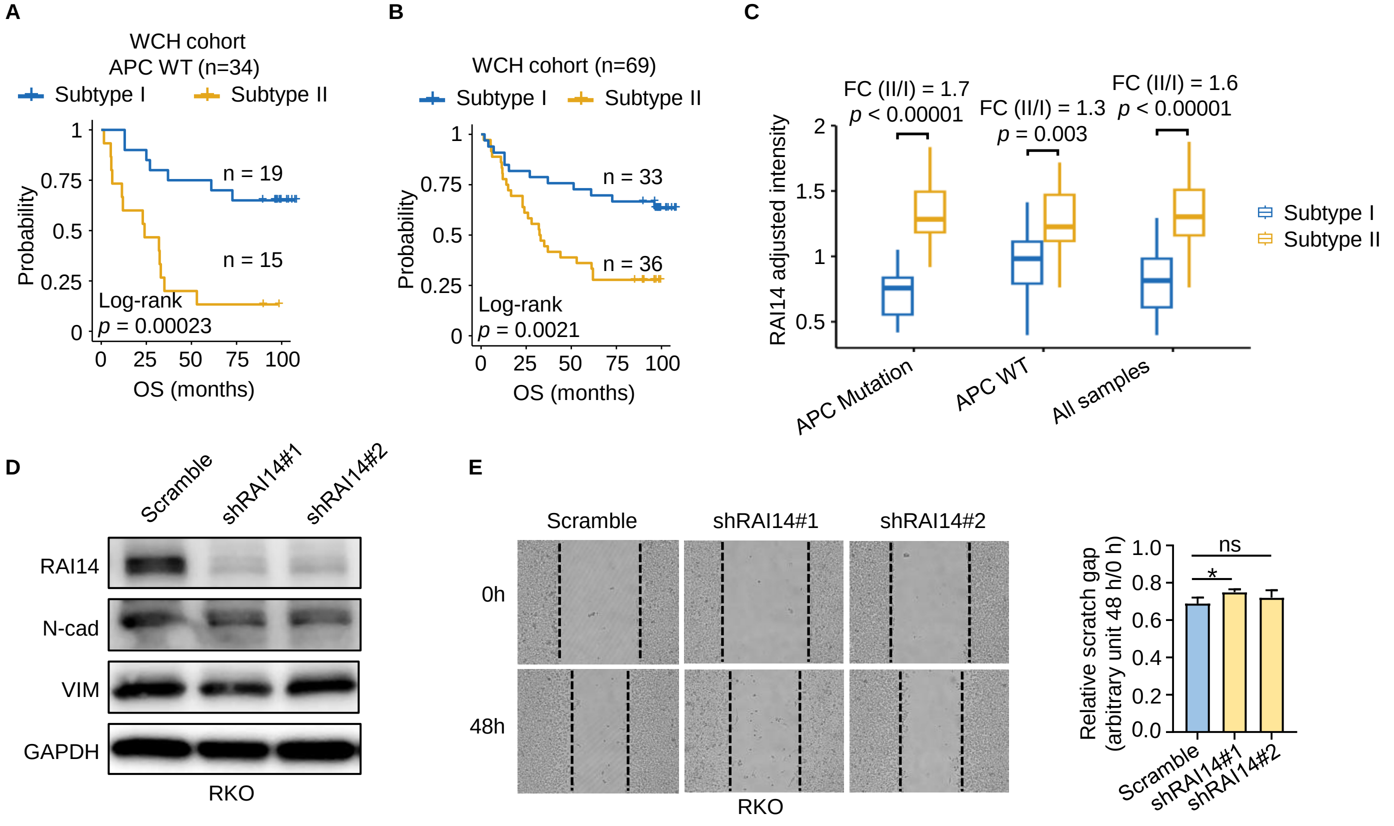

supplemental Fig. S8

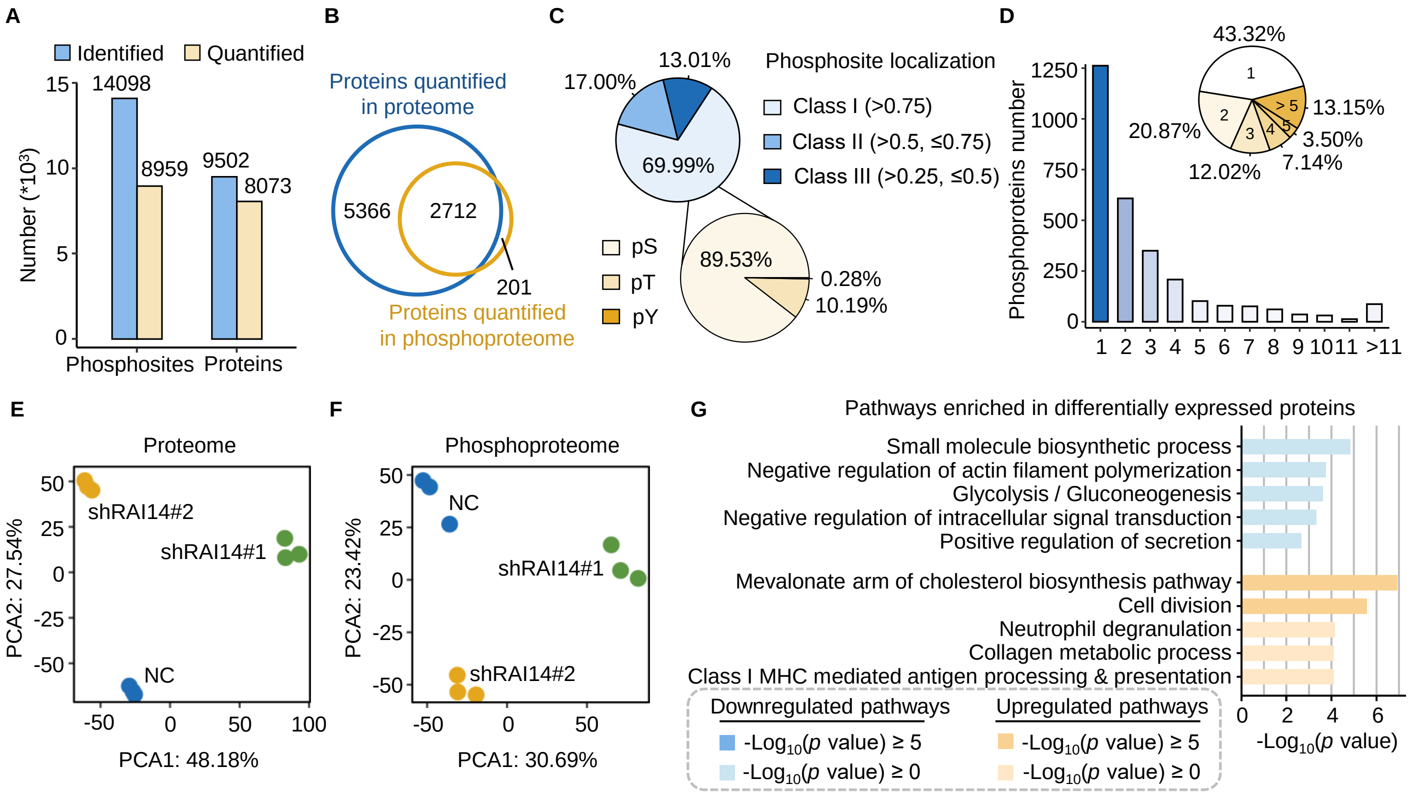

supplemental Fig. S9

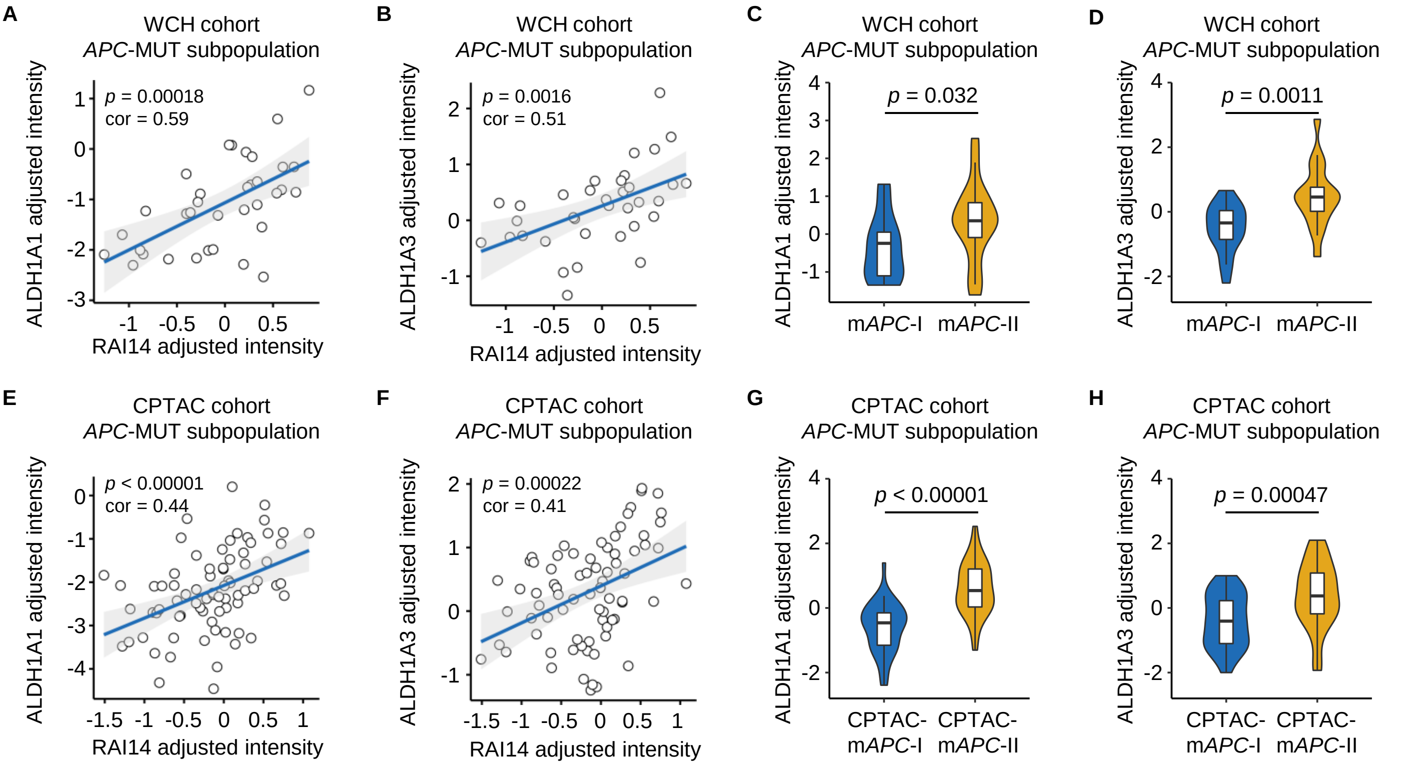

Supplement: Supplemental Figures [file mmc9.pdf]
