# Supplementary material for: Phenotypic Heterogeneity Analysis of APC-Mutant Colon Cancer by Proteomics and Phosphoproteomics Identifies RAI14 as a Key Prognostic Determinant in East Asians and Westerners
Source: Mol Cell Proteomics. 2023 Mar 18;22(5):100532. doi: 10.1016/j.mcpro.2023.100532 (PMC10148045; doi:10.1016/j.mcpro.2023.100532)
Supplement: Supplemental Figures and Tables Caption [file mmc10.docx]

*Supplementary information*

**Phenotypic heterogeneity analysis of *APC*-mutant colon cancer by proteomics and phosphoproteomics identifies RAI14 as a key prognostic determinant in East Asians and Westerners**

Rou Zhang,^1,†^ Meng Hu,^1,†^ Hai-Ning Chen,^2,†^ Xiuxuan Wang,^1,†^ Zhili Xia,^4,†^ Yu Liu,^1^ Rui Wang,^3^ Xuyang Xia,^1^ Yang Shu,^2^ Dan Du,^3^ Wenbo Meng,^4^ Shiqian Qi,^1^ Yuan Li,^5^ Heng Xu,^1^ Zong-Guang Zhou,^2,5^* Lunzhi Dai^1,^*

^1^National Clinical Research Center for Geriatrics and General Practice Medical Center, State Key Laboratory of Biotherapy, West China Hospital, Sichuan University, Chengdu, 610041, China.

^2^Colorectal Cancer Center, Department of General Surgery, West China Hospital, Sichuan University, Chengdu, 610041, China.

^3^West China-Washington Mitochondria and Metabolism Centre, Institutes for Systems Genetics; Advanced Mass Spectrometry Center, Research Core Facility, Frontiers Science Center for Disease-related Molecular Network, West China Hospital, Sichuan University, Chengdu, 610041, China.

^4^The First Clinical Medical College, Lanzhou University, Lanzhou, 730000, China.

^5^Institute of Digestive Surgery, West China Hospital, Sichuan University, Chengdu, 610041, China.

^†^These authors contributed equally to this work.

*Correspondence: lunzhi.dai@scu.edu.cn (Dr. Lunzhi Dai); zhou767@163.com (Dr. Zong-Guang Zhou)

**Supplementary Figure Legends**

**supplemental Fig. S1. Multi-omics characterization of colon cancer tissues.** The experimental design for proteomics, phosphoproteomics and WES is presented (By FigDraw). The number of patients with clinical information and the number of tissues used for each omics study were counted. WES: Whole-exome sequencing.

**supplemental Fig. S2. Quality control (QC) analysis of the proteome.** *A*, Correlation analysis of 18 internal standards to evaluate the robustness of TMT-based quantification in the proteomic study. Top-right half: pie chart shows pairwise comparison of the 18 internal standards. Bottom-left half: pairwise calculation of Pearson’s correlation coefficients between the 18 internal standards. *B*, Correlation analysis of 10 runs of QC samples to evaluate the stability of the machine. Top-right half: elliptic chart shows pairwise comparison of the 10 QC samples. Bottom-left half: pairwise calculation of Pearson’s correlation coefficients between the 10 samples. *C*, t-SNE plot of different batches of samples. *D*, Pearson’s correlation analysis of the replicate samples. *E*, Adjusted protein intensity distribution of each sample. *F*, Cumulative number of identified proteins. *G*, Density distribution of adjusted protein intensity before and after data imputation. Blue represents data before imputation, and yellow represents data after imputation.

**supplemental Fig. S3. QC analysis of the phosphoproteome.** *A*, Cumulative frequency distribution of the coefficient of variations (CVs) (%) of phosphosites for the internal standard samples. *B*, Pearson’s correlation analysis of 6 runs of QC samples to evaluate the stability of the machine. Top-right half: elliptic chart shows pairwise comparison of the 6 QC samples. Bottom-left half: pairwise calculation of Pearson’s correlation coefficients between the 6 QC samples. *C*, t-SNE plot of different batches of samples. *D*, Adjusted abundance distribution of phosphosites for each sample. *E*, Cumulative number of identified phosphosites. *F*, Venn diagram showing the total number of phosphoproteins identified in the proteome. *G*, Pie charts show the localization and distribution of phosphosites. *H*, Density distribution of adjusted phosphosite intensities before and after data imputation. Blue represents data before imputation, and yellow represents data after imputation.

**supplemental Fig. S4. Proteome- and phosphoproteome-based molecular classification of *APC*-MUT colorectal cancer and the differences between the two subtypes.** *A*, Volcano plot showing 523 proteins with favorable prognosis and 179 proteins with unfavorable prognosis in the *APC*-MUT subpopulation of the WCH cohort (log-rank test, *p* < 0.05). Yellow and blue dots represent favorable prognosis and unfavorable prognosis, respectively. Protein intensities in tumors adjusted to those in DNT were used to perform correlation analysis. *B*, Volcano plot shows 136 phosphosites with favorable prognosis and 51 phosphosites with unfavorable prognosis in the *APC*-MUT subpopulation of the WCH cohort (log-rank test, *p* < 0.05). Yellow and blue dots represent the phosphosites that predict favorable and unfavorable prognosis, respectively. Phosphopeptide intensities in tumors adjusted to those in DNT were used to perform correlation analysis. *C*, Heatmap representation of unsupervised clustering of the proteome and phosphoproteome data in the *APC*-MUT subpopulation of the WCH cohort. *D*, The silhouette width of unsupervised clustering based on the CancerSubtypes method in integrated proteome and phosphoproteome data when k = 2 in the WCH cohort. *E*, Heatmap representation of unsupervised clustering of the proteome and phosphoproteome data in the *APC*-MUT subpopulation of the CPTAC cohort. *F*, The silhouette width of unsupervised clustering based on the CancerSubtypes method in integrated proteome and phosphoproteome data when k = 2 in the CPTAC cohort. *G*, Kaplan-Meier curves for patients with CPTAC-m*APC*-I and CPTAC-m*APC*-II subtypes (log-rank test). *H*, Mutation frequency of significantly mutated genes (SMGs) in the m*APC*-I and m*APC*-II subtypes of the WCH cohort. Test method, Fisher’s exact test for comparison, *****p* < 0.0001, ****p* < 0.001, ** *p* < 0.01, * *p* < 0.05. *I*, Mutation frequency of SMGs in CPTAC-m*APC*-I and CPTAC-m*APC*-II subtypes. Test method, Fisher’s exact test for comparison, *****p* < 0.0001, ****p* < 0.001, ** *p* < 0.01, * *p* < 0.05. The mutation frequency of *SMAD4* was higher in the m*APC*-I subtype in the WCH cohort but not in the CPTAC cohort, indicating that SMAD4 is not a prognostic determinant for APC-MUT colon cancer patients in both East Asians and Westerners.

**supplemental Fig. S5. Functional annotation of the m*APC*-II-associated phosphoproteome.** Pathway enrichment analysis of the corresponding proteins of downregulated phosphosites in the m*APC*-II subtype using Metascape. The color of the nodes in the network represents different biological pathway terms. Node size is proportional to the number of input genes that are classified within that term, while node color represents the identity of the cluster to which it belongs. The edges linking terms with a similarity score greater than 0.3 are displayed, with the thickness of the edges representing the degree of similarity between biological pathways.

**supplemental Fig. S6. Potential drug targets for the m*APC*-II subtype.** *A*, Volcano plot showing 641 upregulated and 65 downregulated proteins in the CPTAC-m*APC*-II subtype. Test method, Wilcoxon rank-sum test, with *p* < 0.05 indicating significant changes and cutoff ratio (CPTAC-m*APC*-II/CPTAC-m*APC*-I) > 1.5 or < 0.667. Blue and yellow dots represent significantly differentially expressed proteins in the CPTAC-m*APC*-I and CPTAC-m*APC*-II subtypes, respectively. *B*, GOBP enrichment analysis of proteins with adjusted intensity that were significantly upregulated in the CPTAC-m*APC*-II subtype. *C*, Venn diagram showing the number of overlapping proteins upregulated in the WCH and CPTAC cohorts. Sixty-one overlapping proteins are known drug targets with approved drugs or drugs in clinical trials. *D*, The statistics of the relative protein expression of the 61 drug targets in the m*APC*-II and m*APC*-II subtypes of the WCH and CPTAC cohorts, respectively (Wilcoxon rank-sum test, *p* < 0.05). *E*, Seventeen metastasis-associated proteins were upregulated in m*APC*-II tumors. Test method, Wilcoxon rank-sum test, with *p* < 0.05 indicating significant changes and cutoff ratio (m*APC*-II/m*APC*-I) > 2. The age, gender, TNM stage, survival event, OS and mutation load were annotated above the heatmap. The heatmap depicts the adjusted intensity of proteins with log_2_-transformation. The corresponding subcellular location (left), catalytic activity (left) and clinical drugs (right) are listed.

**supplemental Fig. S7. The relationship between RAI14 and APC.** *A*, *B*, Kaplan-Meier curves for colon cancer patients in the subtype I and subtype II from the APC-WT subpopulation (*A*) and the total 69 patients (*B*) in the WCH cohort. Test methods, log-rank test. *C*, Boxplots showing the comparison of RAI14 adjusted intensity between subtype I and subtype II in three comparisons. *D*, Western blot analyses demonstrating the effects of RAI14 on the expression of EMT markers in APC-WT colon cancer cell line RKO. *E*, Wound healing assays illustrating cell migration of RKO after 48 h in response to RAI14 knockdown. Test method, Student’s t test, *****p* < 0.0001, ****p* < 0.001, ** *p* < 0.01, * *p* < 0.05, for indicated comparisons.

**supplemental Fig. S8. QC analysis of RAI14-dependent proteomics and phosphoproteomics profiling.** *A*, The numbers of identified and quantified proteins and phosphosites in DLD-1 cells after RAI14 knockdown. *B*, Venn diagram showing the number of phosphoproteins identified in the proteome. *C*, Pie charts of phosphosite localization and distribution. *D*, Statistics of the number of phosphosites in a protein. *E*,*F*, Principal component analysis (PCA) of proteome (*E*) and phosphoproteome (*F*) data in 9 DLD-1 cell samples. Blue dots represent scramble cell samples, green dots represent RAI14-knockdown cell samples by shRAI14#1 plasmid, and yellow dots represent RAI14-knockdown cell samples by shRAI14#2 plasmid. *G*, Pathway enrichment analysis of downregulated and upregulated proteins using Metascape. Yellow represents upregulated pathways, and blue represents downregulated pathways.

**supplemental Fig. S9**. **The expression correlation between RAI14 and enzymes associated with RA biosynthesis in colon cancer.** *A*,*B*, Scatterplots describing the Pearson’s correlation coefficients of ALDH1A1 (*A*) and ALDH1A3 (*B*) correlated with RAI14 in *APC*-MUT patients of the WCH cohort. *C*,*D*, Violin plot illustrating the expression comparison of ALDH1A1 (*C*) and ALDH1A3 (*D*) in m*APC*-I and m*APC*-II patients in the WCH cohort. *E*,*F*, Scatterplots describing the Pearson’s correlation coefficients of ALDH1A1 (*E*) and ALDH1A3 (*F*) correlated with RAI14 in *APC*-MUT patients of the CPTAC cohort. *G*,*H*, Violin plot illustrating the expression comparison of ALDH1A1 (*G*) and ALDH1A3 (*H*) in m*APC*-I and m*APC*-II patients in the CPTAC cohort.

**Supplementary Table Legends**

**supplemental Table S1.** Clinicopathologic characteristics and prognosis information of the WCH cohort and CPTAC cohort, a summary of the included publicly available clinical cohorts, and TMT-labeled information.

**supplemental Table S2.** Genomics data of the WCH cohort and included public clinical cohorts, related to **Fig. 1**.

**supplemental Table S3.** The survival information of colon cancer patients in m*APC-I* and m*APC*-II subtypes, related to **Fig. 2**.

**supplemental Table S4.** Identified proteins and phosphosites, and differentially expressed proteins and phosphosites in m*APC*-I and m*APC*-II determined by Wilcoxon rank-sum test, related to **Fig. 3.**

**supplemental Table S5.** Discriminative signatures of the *APC*-MUT subpopulation, related to **Fig. 4**.

**supplemental Table S6.** Proteomic and phosphoproteomic changes in response to RAI14 silencing, related to **Fig. 6**.

**supplemental Table S7.** RAI14-dependent variables and clinicopathologic characteristics, related to **Fig. 7**.

**supplemental Table S8.** Summary data related to Supplementary Figures.
